# Rash associated with epidermal growth factor receptor inhibitors: a systematic review and meta-analysis of 194 randomized controlled trials

**DOI:** 10.3389/fimmu.2026.1814337

**Published:** 2026-08-21

**Authors:** Fangmin Zhao, Ruiyi Qiu, Yi Liu, Gaochenxi Zhang, Shuyi Chen, Qijin Shu

**Affiliations:** 1 Department of Nutrition, The First Affiliated Hospital of Zhejiang Chinese Medical University (Zhejiang Provincial Hospital of Chinese Medicine), Hangzhou, Zhejiang, China; 2 Department of Oncology, The First Affiliated Hospital of Zhejiang Chinese Medical University (Zhejiang Provincial Hospital of Chinese Medicine), Hangzhou, Zhejiang, China

**Keywords:** adverse event, cutaneous, epidermal growth factor receptor inhibitors, meta-analysis, rash

## Abstract

**Background:**

Rashes in patients treated with epidermal growth factor receptor inhibitors (EGFRIs) are closely related to better clinical outcomes. Multiple randomized controlled trials have evaluated rashes caused by EGFRIs. We conducted a meta-analysis of patients with tumors receiving EGFR-targeted therapy to determine the incidence and relative risk of rashes associated with these drugs.

**Methods:**

Four databases (PubMed, Web of Science, Embase, and Cochrane) were systematically searched to find randomized controlled trials reporting the incidence of rash associated with EGFRIs from the time of database construction to May 15, 2025. Meta-analysis was used to calculate the total incidence and relative risk (RR) of all grades or each grade of rash.

**Results:**

A total of 65,116 patients from 194 studies were included. The pooled incidences of all-grade rash and high-grade rash associated with EGFRIs were 52.30% (95% CI: 48.70%-55.80%) and 9.50% (95% CI: 8.70%-10.30%), respectively. The pooled RR suggested that EGFRIs were associated with a significantly increased risk of rash. Subgroup analysis and regression analysis showed that Asian populations, EGFR monoclonal antibodies (EGFR mAbs), phase III clinical trials, and EGFRI combination therapy may increase the occurrence of rash, whereas Aumolertinib had a significantly lower RR of rash. The type of tumor and the year of publication had no significant effect on the incidence of rash. The trim-and-fill method indicated that the results were reliable.

**Conclusions:**

EGFRIs have a high possibility of increasing the risk of rashes. Prevention and early treatment of rashes could be performed earlier for Asian patients who use EGFR mAbs in combination with radiotherapy and chemotherapy.

**System Review Registration:**

https://www.crd.york.ac.uk/PROSPERO/view/, identifier CRD420251039464.

## 1 Introduction

Epidermal growth factor receptor (EGFR) initiates downstream signaling pathways such as the Ras/RAF/MEK and PI3K/AKT/mTOR pathways, which participate in regulating cell proliferation, migration, survival and antiapoptotic responses. Many solid tumors, such as non-small cell lung cancer (NSCLC), colorectal cancer, and head and neck squamous cell carcinoma, exhibit abnormal expression of EGFR. Therefore, EGFR has become an effective target for the development of antitumor drugs (1). Epidermal growth factor receptor inhibitors (EGFRIs), including tyrosine kinase inhibitors (TKIs, such as Gefitinib, Erlotinib, and Osimertinib) and monoclonal antibodies (mAbs, such as Cetuximab, and Panitumumab), have completely transformed the landscape of tumor treatment. For instance, in NSCLC patients, compared with chemotherapy, EGFR-TKIs can significantly prolong the disease-free survival of patients with EGFR mutations by 46.95 months *vs* 22.11 months (2). These therapies have been incorporated into the standard treatment plans of international guidelines, such as the National Comprehensive Cancer Network (NCCN) (3), and have been approved by the U.S. Food and Drug Administration, fully demonstrating their wide application value in the treatment of various tumors (4–8).

Although EGFRIs have shown significant clinical benefits, their therapeutic efficacy is often accompanied by unique toxic reactions. Skin adverse events (the most typical of which is EGFRIs-related rash) are the most common characteristic toxicity of this type of drug, affecting up to 90% of patients (9). This type of rash usually presents with main symptoms such as erythema papules, pustules, dry skin, pruritus and desquamation (9), which not only significantly reduce the patient’s quality of life, as physical discomfort and psychological distress often interfere with the patient’s social activities and emotional stability (10) but can also can lead to drug reduction or treatment suspension, which can weaken treatment compliance and thereby affect clinical outcomes (11) in 10% to 20% of patients with severe rashes (grade ≥3). Moreover, rash management requires additional medical resources (such as topical glucocorticoids, antibiotics, and frequent outpatient follow-ups), leading to an increase in medical expenses (12). Interestingly, the latest research suggests that the occurrence of rashes may be associated with improved treatment response: patients with rashes tend to have better progression-free survival (HR: 0.55, 95% CI: 0.33-0.92, *P* = 0.020) and overall survival (HR: 0.64, 95% CI: 0.46-0.89, *P* = 0.018) *(*12–14). Even so, this association does not reduce the necessity of actively managing the severity of the rash. Achieving a balance between efficacy and tolerance remains crucial.

Although EGFRIs-related rashes have significant clinical significance, the literature still has obvious limitations. First, the incidence of rashes reported by different studies varies significantly: the incidence of all-grade rashes ranges from 11.6% to 92.8% (15, 16), and that of severe rashes (grade ≥3) varies from 1.0% to 33.8% (17, 18). This inconsistency may stem from differences in research design, patient cohorts, and types of EGFRIs used, which makes it difficult to determine the true burden of the rash (especially severe rash events that affect treatment decisions). Second, relatively few systematic studies on the factors influencing the risk of rashes exist. These factors include tumor type, type of EGFRIs, treatment regimens (such as comparisons between monotherapy and combination therapy), and patient characteristics (such as race and age) (19), which are key to formulating individualized prevention strategies, but the statistical power of a single trial is insufficient to conduct meaningful subgroup analyses. The third deficiency is reflected in the rapid development of the field: new drugs (such as Necitumumab, Lazertinib) (20, 21) and updated treatment regimens are constantly emerging, but there is currently no latest meta-analysis integrating data from recent randomized controlled trials (RCTs). This knowledge gap makes it impossible for clinicians to provide precise risk assessment and makes it difficult to guide patients in individualized rash management.

This systematic review and meta-analysis aims to fill the aforementioned gap by conducting a quantitative synthesis of RCT data, calculating the combined incidence of all and each grade of rash associated with EGFRIs, assessing the relative risk (RR) of rash under different EGFRIs, tumor types, and treatment strategies through subgroup analysis, and exploring the sources of heterogeneity. By integrating high-quality evidence, this analysis comprehensively presents the characteristics of rash risk, thereby helping clinicians identify high-risk patients and implement active intervention measures.

## 2 Methods

Our systematic reviews and meta-analyses were reported in accordance with the preferred reporting items of the systematic reviews and meta-analyses (PRISMA) guidelines and have been registered with PROSPERO (CRD420251039464).

### 2.1 Search strategy

We systematically searched the PubMed, Embase, Cochrane and Web of Science databases on May 15, 2025, without language restrictions, as well as the reference lists of all relevant articles and reviews. The search strategy is based on the PICOS principle and uses a combination of medical subject headings (MeSH) terms and entries. These include EGFRIs (Gefitinib, Erlotinib, Icotinib, Afatinib, Dacomitinib, Osimertinib, Aumolertinib, Lazertinib, Cetuximab, Panitumumab, Necitumumab, Nimotuzumab, etc.), rashes, and RCTs (**Supplementary Table 1**-**4**). Repeated references have been removed.

### 2.2 Inclusion criteria and study selection

The two authors (FMZ and RYQ) first screened the titles and abstracts of all the articles obtained and carefully evaluated whether the full texts of the selected studies met the criteria. Disagreements were resolved through discussions with a third reviewer (YL). We selected studies that met the following conditions: (1) cancer patients, (2) participants who were assigned to receive EGFRIs treatment (alone or in combination, at any dose or frequency) or control treatment, (3) available safety data for simple skin rashes, and (4) prospective, randomized, controlled phase II and III trials. We excluded comments, protocols, meta-analyses, reviews, safety data of other dermatologic types (psoriasis, vitiligo, herpes), or studies that were published only in abstract form.

### 2.3 Data extraction and quality assessment

One author (RYQ) used a predefined data extraction form to extract data from each study. Another author (FMZ) checked the extracted data from each study to ensure that all the data were correctly extracted. The core baseline and outcome data, including the first author, publication year, region/country, study type, tumor type, number of participants included and their ages, male gender ratio, follow-up time, intervention measures, and study results, were extracted. The primary outcome measure was the incidence of all grades and each grade of rash. The quality of each eligible study was evaluated via the Cochrane Risk of Bias 2 (RoB2) tool to assess the risk of bias in the included studies. Two reviewers independently evaluated the quality of the included studies. Any doubts were resolved through discussion and further review.

### 2.4 Statistical analysis

We conducted a fixed and random effects meta-analysis via Stata version 18. The statistical heterogeneity was reported as the *Q* statistic and the *I^2^* statistic (22). When *P*>0.05 and *I^2^* < 50%, the homogeneity of the studies was good, and the fixed effect model was used to combine the effect sizes; when *P ≤* 0.05 or *I^2^*≥50%, the homogeneity of the studies was poor, and the random effect model was used for correction. When significant heterogeneity was found among the studies, we conducted subgroup analyses to examine the possible sources of heterogeneity. The subgroup analysis applied separate random-effects models with DerSimonian-Laird (DL) weights. To control the impact of confounding factors and quantify each variable on RR, we conducted a multivariate meta-regression analysis. The included covariates were consistent with those analyzed in the subgroup analysis. Meta-regression was fitted as a random-effects model with restricted maximum likelihood (REML) estimation of between-study variance (τ²). If *P*> 0.05 for a certain variable, it is considered that the variable has no independent effect after adjusting for other factor. We also conducted a network meta-analysis for the rash RR of EGFRIs monotherapy, adopting a random effects consistency model (23). Ranking probabilities were estimated via the surface under the cumulative ranking curve (SUCRA) (24). We set the criterion of including only trials of EGFRIs monotherapy to ensure transitivity. We employed the chi-square test to evaluate global inconsistency. To calculate the local inconsistency (25), we evaluated node inconsistency by the node splitting method and the inconsistency of the closed loop using the ROutside/ROinside ratio.

Publication bias was evaluated by visually observing the funnel plot. The formal statistical assessment of funnel plot asymmetry also employed Egger’s regression asymmetry test and Begg’s test (26), with *P*>0.05 and a symmetrical funnel plot suggesting no potential bias; otherwise, we use trim-and-fill methods to detect the impact of possibly missing studies on the overall effect. We also conducted a sensitivity analysis, in which each study was excluded in sequence to check the influence of that study on the overall estimation.

## 3 Results

### 3.1 Eligible studies and patient characteristics

**Figure 1:**
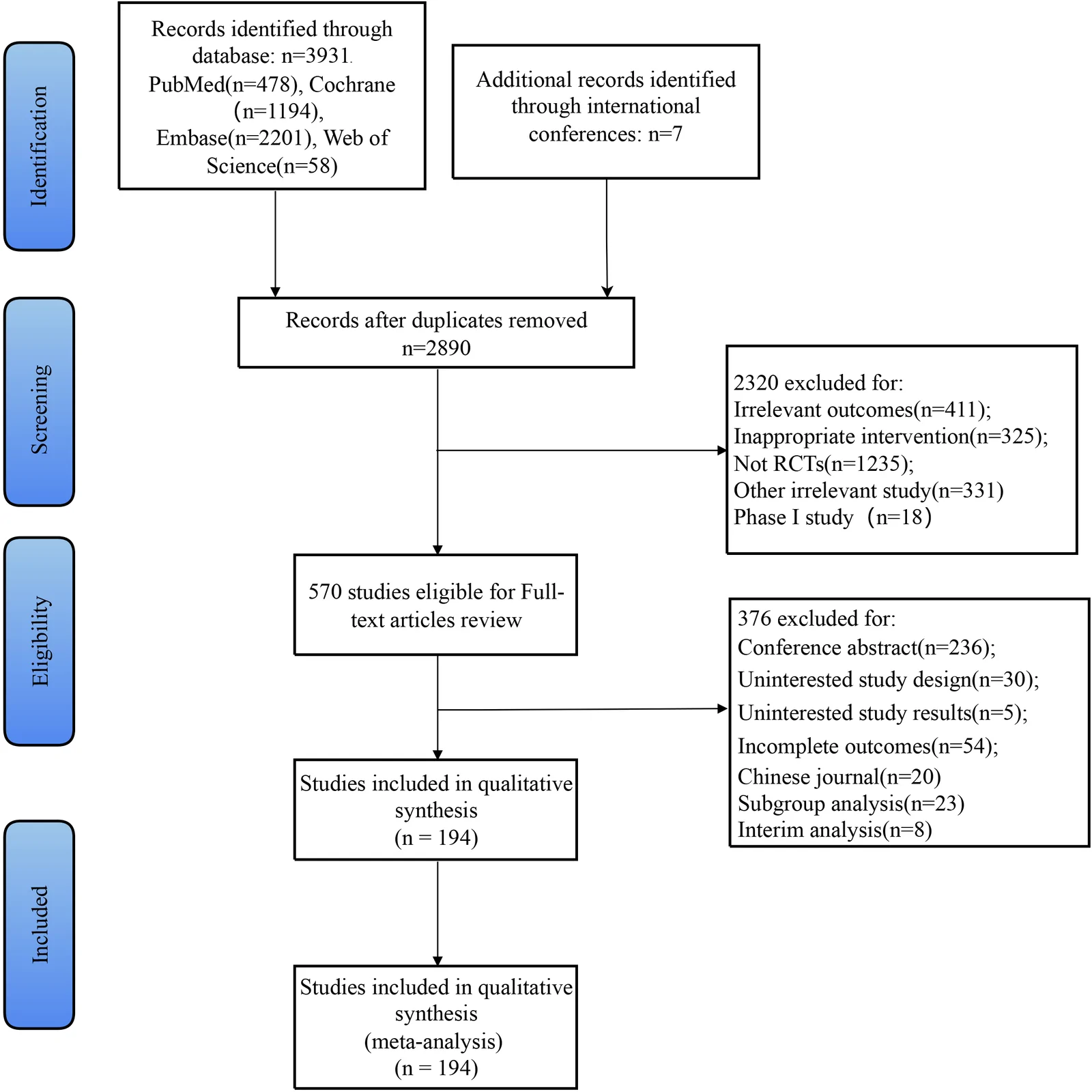
Research selection process for randomized controlled trials (RCTs).

A total of 3938 studies were retrieved (**Figure 1**). After removing duplicate content and screening the titles and abstracts, the search identified 2890 publications. Among them, 2696 were excluded because they did not meet the inclusion criteria. A total of 194 studies (containing 65,116 participants) were included in our analysis. The baseline characteristics of the included studies are presented in **Supplementary Table 5**.

Among the 194 included studies, 183 articles reported that patients exhibiting rash were diagnosed and graded on the National Cancer Institute’s Common Terminology Criteria for Adverse Events (NCI-CTCAE). The rash diagnosis in the five articles was made by researchers based on clinical characteristics after conducting physical examinations on patients, and supported by histopathology when necessary. Six articles did not report their diagnostic criteria for rash. All articles were published between 2003 and 2025 (with 33 studies published in 2020 and later). Forty-one studies originated from Asia, and 62 studies came from around the world. Ninety-five studies included only patients with non-small cell lung cancer, 23 studies included only patients with head and neck cancer, 46 studies included only patients with colorectal cancer, 8 studies included only patients with breast cancer, and the remaining tumor types included 2 cases of biliary tract cancer, 1 case of cervical cancer, 2 cases of gastric cancer, 2 cases of glioblastoma, 1 case of hepatocellular carcinoma, 3 cases of esophagogastric cancer, 1 case of ovarian cancer, 6 cases of pancreatic cancer, 1 case of prostate cancer, 2 cases of renal cell cancer, and 2 cases of urothelial cancer. One hundred sixty-two studies (53,152 participants) focused on advanced (stage III-IV, advanced, recurrent or metastatic) tumors. Subgroup grouping: Sixty-three studies involving 24,853 patients who received EGFRI monotherapy (the control group included 18 EGFRIs, 15 blank controls, and 25 patients receiving chemotherapy) and 131 studies involving 38,900 patients who received EGFRI combination therapy (the control group included 34 patients receiving EGFRI monotherapy, 21 patients receiving EGFRI combination therapy, 76 patients receiving chemotherapy and other treatments) were included.

### 3.2 The quality of the included studies

All included studies were assessed via the RoB2 tool. Among them, 59 studies (30.41%) were judged to be at low risk of bias, 4 studies (2.06%) at high risk of bias, and the remaining 131 studies (67.53%) at unclear risk of bias. Although the number of high-risk studies is small, the vast majority of them have a relatively high risk of uncertainty because the method of randomization as well as the number and reason for deletion is unclear. The overall results of the risk of bias assessment are presented in **Supplementary Figure 1**. Furthermore, we conducted a sensitivity analysis to explore the potential impact of high-risk and unclear bias studies on the pooled effect size.

### 3.3 The incidence of rash

The clinical manifestations of the rashes included eczema, acneiform dermatitis, maculopapular rash, and erythema. For all-grade rash, the incidence ranged from 0 to 98.70%, with a cumulative incidence (events/total events) of 22646/42584 (53.18%), and the pooled incidence (weighting the research data through meta-analysis on the premise of respecting heterogeneity) was 52.30% (95% CI: 48.70%-55.80%, *I^2^* = 98.80%, *P* < 0.001) (**Table 1**, **Supplementary Figure 2**). The cumulative incidence of grades 1–2 rashes was 9277/16126 (57.53%), and the pooled incidence estimate was 57.20% (95% CI: 54.10%-60.20%, *I^2^* = 93.90%, *P* < 0.001) from 71 reports. The incidence of grades ≥3 rashes was 3987/41299 (9.65%), and the pooled incidence estimate was 9.50% (95% CI: 8.70%-10.30%, *I^2^* = 90.90%, *P* < 0.001) from 181 reports (**Table 1**).

**Table 1:** Incidence and relative risk of rash with EGFRIs according to nation, study phase, tumor type, therapeutic regimen, EGFRI type and publication year.

Groups	All-grade								G1-2								G≥3							
	No. of studies	Summary incidence(95% CI)%	*P*- value	*I^2^*(%)	RR (95% CI)	*P*- value	*I^2^* (%)	*P*- interaction	No. of studies	Summary incidence(95% CI)%	*P*- value	*I^2^* (%)	RR (95% CI)	*P*- value	*I^2^* (%)	*P*- interaction	No. of studies	Summary incidence(95% CI)%	*P*- value	*I^2^* (%)	RR (95% CI)	*P*-value	*I^2^* (%)	*P*-interaction
**Overall**	194	52.30 (48.70-55.80)	<0.001	98.80	2.62(2.37-2.90)	<0.001	95.70		71	57.20 (54.10-60.20)	<0.001	93.90	2.38 (1.99-2.84)	<0.001	96.00		184	9.50 (8.70-10.30)	<0.001	90.90	3.69 (2.99-4.55)	<0.001	74.50	
**Nation**								**0.42**								**0.099**								**0.764**
Asian	41	60.80 (54.20-67.50)	<0.001	98.10	2.55 (2.12-3.06)	<0.001	95.40		13	65.60 (59.80-71.40)	<0.001	87.60	1.77 (1.36-2.32)	<0.001	91.50		40	8.50 (6.80-10.20)	<0.001	90.90	3.30 (2.04-5.35)	<0.001	69.40	
No-Asian	91	51.20 (45.80-56.60)	<0.001	98.40	2.90 (2.44-3.45)	<0.001	94.20		38	56.20 (52.30-60.20)	<0.001	89.60	2.81 (2.03-3.90)	<0.001	95.60		84	12.40 (10.90-14.00)	<0.001	88.30	4.02 (2.90-5.60)	<0.001	74.60	
Multinational	62	48.60 (42.90-54.30)	<0.001	99.10	2.48 (2.07-2.97)	<0.001	96.90		20	53.30 (47.60-59.10)	<0.001	96.70	2.24 (1.64-3.05)	<0.001	97.40		60	7.10 (6.20-8.00)	<0.001	90.50	3.52 (2.51-4.96)	<0.001	76.70	
**Study phase**								<0.001								<0.001								<0.001
Phase II	108	51.40 (41.00-56.80)	<0.001	98.40	1.52 (1.39-1.67)	<0.001	84.70		38	58.30 (54.90-61.80)	<0.001	81.40	1.31 (1.12-1.52)	0.001	84.10		103	9.90 (8.70-11.00)	<0.001	78.60	2.01 (1.60-2.52)	<0.001	49.40	
PhaseIII	86	53.50 (48.50-58.50)	<0.001	99.10	4.02 (3.38-4.78)	<0.001	97.50		33	56.00 (51.30-60.80)	<0.001	96.80	3.87 (2.86-5.24)	<0.001	97.80		81	9.10 (8.10-10.20)	<0.001	94.80	6.74 (4.78-9.49)	<0.001	83.10	
**Tumor type**								0.008								0.02								**0.752**
NSCLC	95	54.60 (49.80-59.50)	<0.001	98.90	2.52 (1.99-2.55)	<0.001	96.30		33	59.60 (55.00-64.30)	<0.001	95.60	1.74 (1.41-2.15)	<0.001	96.00		93	7.80 (6.90-8.70)	<0.001	90.50	3.22 (2.42-4.27)	<0.001	75.30	
Colorectal Cancer	46	50.30 (42.60-58.00)	<0.001	99.10	3.32 (2.61-4.23)	<0.001	96.10		15	60.70 (55.80-65.60)	<0.001	87.20	2.96 (1.76-5.00)	<0.001	97.30		46	13.20 (11.30-15.20)	<0.001	92.90	4.15 (2.72-6.33)	<0.001	80.10	
Head and Neck Cancer	23	55.20 (44.80-65.60)	<0.001	98.00	2.81 (1.92-4.12)	<0.001	94.50		10	60.10 (54.50-65.80)	<0.001	76.70	3.58 (2.16-5.91)	<0.001	90.30		20	10.90 (8.10-13.80)	<0.001	84.20	5.10 (2.25-11.56)	<0.001	66.90	
Breast Cancer	8	39.60 (29.80-49.50)	<0.001	87.80	3.06 (1.62-5.77)	0.001	80.70		4	40.00 (35.70-44.30)	<0.001	6.00	2.43 (0.96-6.17)	**0.061**	88.10		6	5.80 (3.10-8.50)	<0.001	47.40	3.20 (1.04-9.89)	0.043	13.70	
Other*	22	46.60 (36.80-56.30)	<0.001	97.10	4.45 (2.70-7.34)	<0.001	92.20		9	48.10 (39.60-56.50)	<0.001	91.40	4.19 (1.79-9.82)	0.001	95.10		19	7.80 (5.90-9.80)	<0.001	70.10	4.06 (2.20-7.49)	<0.001	47.20	
**Therapeutic regimen**								<0.001								<0.001								<0.001
EGFRI monotherapy vs other	63	54.20 (49.00-59.30)	<0.001	98.30	2.61 (2.18-3.12)	<0.001	96.00		22	56.10 (50.70-61.40)	<0.001	94.30	2.14 (1.63-2.81)	<0.001	95.20		58	7.20 (6.10-8.20)	<0.001	91.60	3.81 (2.52-5.75)	<0.001	76.00	
EGFRI combination vs other	76	50.90 (44.00-57.80)	<0.001	99.10	7.98 (6.18-10.31)	<0.001	93.00		25	59.70 (54.20-65.10)	<0.001	93.90	7.27 (5.34-9.88)	<0.001	86.10		71	12.30 (10.70-14.00)	<0.001	88.90	13.70 (9.40-19.97)	<0.001	57.10	
EGFRI combination vs EGFRI monotherapy	34	55.30 (48.30-62.40)	<0.001	98.40	1.02 (0.97-1.07)	**0.504**	45.00		16	54.30 (47.90-60.80)	<0.001	94.70	0.91 (0.84-0.99)	0.033	45.50		33	0.90 (7.50-10.40)	<0.001	86.50	1.37 (1.08-1.74)	0.01	46.00	
EGFRI combination vs EGFRI combination	21	46.50 (36.20-56.80)	<0.001	98.90	1.02 (0.93-1.12)	**0.689**	61.50		8	60.50 (54.20-66.80)	<0.001	81.00	1.02 (0.90-1.16)	**0.746**	42.90		22	9.30 (7.40-11.20)	<0.001	83.50	1.05 (0.76-1.44)	0.778	42.00	
**Drug type**								<0.001								<0.001								0.007
TKI	116	54.40 (50.00-58.70)	<0.001	98.60	2.08 (1.87-2.31)	<0.001	94.80		45	56.80 (52.80-60.70)	<0.001	94.50	1.65 (1.40- 1.94)	<0.001	93.40		110	7.70 (6.80-8.50)	<0.001	89.30	2.87 (2.23-3.71)	<0.001	70.60	
mAb	78	49.30 (43.40-55.20)	<0.001	99.00	3.94 (3.18-4.87)	<0.001	96.40		26	58.30 (54.00-62.60)	<0.001	91.00	4.43 (2.91-6.77)	<0.001	97.10		74	12.20 (10.70-13.60)	<0.001	91.10	5.30 (3.69-7.61)	<0.001	78.20	
**EGFRI**								<0.001								<0.001								0.002
Gefitinib	27	53.90 (47.80-60.10)	<0.001	96.40	2.21 (1.64-2.98)	<0.001	94.80		10	61.80 (56.30-67.40)	<0.001	82.30	1.16 (0.86-1.58)	**0.337**	86.90		25	4.40 (3.20-5.60)	<0.001	81.90	2.32 (1.23-4.38)	0.009	64.50	
Erlotinib	57	52.90 (45.80-59.90)	<0.001	99.10	2.07 (1.80-2.39)	<0.001	94.40		21	54.50 (48.10-60.80)	<0.001	95.60	1.47 (1.17-1.83)	0.001	91.20		57	9.30 (8.00-10.60)	<0.001	88.50	3.10 (2.23-4.29)	<0.001	72.70	
Icotinib	3	27.60 (11.60-43.70)	0.001	93.30	1.31 (0.61-2.82)	**0.485**	83.50		–	–	–	–	–	–	–		3	7.40 (-6.7-21.5)	**0.303**	95.70	2.64 (0.14-50.00)	**0.518**	77.80	
Afatinib	18	70.40 (64.90-75.90)	<0.001	92.40	2.55 (1.87-3.48)	<0.001	95.60		9	61.70 (54.30-69.10)	<0.001	92.40	3.08 (1.77-5.37)	<0.001	96.60		16	8.50 (6.40-10.60)	<0.001	76.50	3.05 (1.51-6.17)	0.002	71.20	
Dacomitinib	3	41.60 (18.60-64.70)	<0.001	98.60	2.31 (0.73-7.28)	**0.154**	97.00		2	45.00 (40.00-50.10)	<0.001	59.30	2.53 (0.39-16.57)	**0.332**	98.30		3	7.00 (3.90-10.10)	<0.001	76.90	7.95 (1.33-47.70)	0.023	68.60	
Osimertinib	4	54.60 (43.60-65.50)	<0.001	84.80	1.43 (0.77-2.66)	**0.257**	92.80		2	57.40 (52.70-62.20)	<0.001	0.00	0.89 (0.68-1.18)	**0.413**	66.90		4	0.90 (0.20-1.60)	0.011	0.00	0.35 (0.08-1.59)	**0.174**	33.10	
Aumolertinib	1	23.40 (17.70-29.00)	<0.001	–	0.56 (0.42-0.75)	<0.001	–		–	–	–	–	–	–	–		–	–	–	–	–	–	–	
Lazertinib	1	36.20 (29.50-43.00)	<0.001	–	0.99 (0.76-1.29)	**0.947**	–		–	–	–	–	–	–	–		–	–	–	–	–	–	–	
Cetuximab	55	47.60 (40.60-54.70)	<0.001	99.10	3.88 (2.99-5.05)	<0.001	96.40		20	56.30 (50.90-61.70)	<0.001	92.60	4.38 (2.67-7.18)	<0.001	97.00		52	12.20 (10.40-13.90)	<0.001	91.10	5.92 (3.72-9.42)	<0.001	79.10	
CMAB009	1	66.90 (61.80-71.90)	<0.001	–	12.26 (6.47-23.24)	<0.001	–		–	–	–	–	–	–	–		1	6.50 (3.90-9.10)	<0.001	–	10.74 (1.46-78.99)	0.02		
Panitumumab	17	54.40(43.60-65.20)	<0.001	97.90	3.50 (2.37-5.17)	<0.001	95.10		5	63.80 (59.90-67.60)	<0.001	24.20	4.30 (1.56-11.85)	0.005	96.80		16	13.50 (10.20-16.80)	<0.001	91.10	2.75 (1.60-4.74)	<0.001	69.30	
Necitumumab	4	56.80 (28.70-85.00)	<0.001	99.10	5.15 (3.05-8.72)	<0.001	81.30		1	69.10 (65.20-73.00)	<0.001	–	7.06 (5.43-9.17)	<0.001	–		4	8.10 (2.40-13.80)	0.006	90.60	19.91 (6.81-58.18)	<0.001	0.00	
Nimotuzumab	1	25.00 (11.60-38.40)	<0.001	–	5.25 (1.23-22.50)	0.026	–		–	–	–	–	–	–	–		1	–	–	–	–	–	–	
Gefitinib or erlotinib	1	55.40 (10.80-99.90)	0.015	98.80	1.82 (1.15-2.87)	0.01	–		1	30.90 (22.70-39.10)	<0.001	–	1.73 (1.09-2.74)	0.02	–		1	1.60 (-0.60-3.90)	**0.154**	–	5.00 (0.24-103.09)	**0.297**	–	
AZD8931	1	2.00 (-0.20-40.20)	**0.053**	–	7.44 (0.42-132.96)	**0.173**	–		–	–	–	–	–	–	–		1	20.00 (-0.20-40.20)	**0.053**	–	7.44 (0.42-132.96)	**0.173**	–	
**Published year**								0.01								0.045								**0.107**
More earlier	161	52.20 (48.30-56.10)	<0.001	98.90	2.75 (2.45-3.08)	<0.001	96.00		54	57.20 (53.90-60.60)	<0.001	94.30	2.57 (2.09-3.16)	<0.001	96.70		156	9.60 (8.80-10.40)	<0.001	91.10	3.93 (3.11-4.96)	<0.001	75.40	
Within 5 years	33	52.40 (44.10-60.00)	<0.001	98.60	2.03 (1.66-2.48)	<0.001	92.60		17	56.80 (49.20-64.50)	<0.001	92.10	1.75 (1.28-2.39)	0.001	90.20		28	8.80 (7.00-10.60)	<0.001	89.10	2.55 (1.60-4.08)	<0.001	65.30	

CI, confidence interval; NSCLC, non-small cell lung cancer; * for all-grade: 2 Biliary tract cancer, 1 Hepatocellular Carcinoma, 1 Cervical cancer, 3 Oesophago-gastric cancer, 2 Gastric cancer, 2 Glioblastoma, 6 Pancreatic cancer, 1 Prostate cancer, 2 Renal Cell Cancer, 2 Urothelial cancer; * for G1-2: 1 Prostate cancer, 2 Pancreatic cancer, 1 Gastric cancer, 2 Oesophago-gastric cancer, 1 Cervical cancer, 1 Biliary tract cancer, 1 Urothelial cancer; *for G≥3: 2 Biliary tract cancer, 1 Cervical cancer, 2 Gastric cancer, 2 Glioblastoma, 1 Hepatocellular Carcinoma, 2 Oesophago-gastric cancer, 6 Pancreatic cancer, 2 Pancreatic cancer, 1 Urothelial cancer; 13 studies were not estimable for G≥3, of which the events of both groups were 0. Values with *P* > 0.05 were in bold.

### 3.4 Relative risk analysis

The relevant rash risk of 194 studies was calculated via the random effects model (*I^2^* = 95.70%). Compared with the control, EGFRIs significantly increased the risk of all-grade rashes, with a relative risk of 2.62 (95% CI: 2.37–2.90, *P* < 0.001) (**Table 1**, **Supplementary Figure 3**). For low-grade rashes (G1-2), the RR of EGFRIs according to the random effects model (*I^2^* = 96.00) was 2.38 (95% CI: 1.99-2.84, *P* < 0.001). In the combined 181 studies, EGFRIs also increased the risk of high-grade rashes (G≥3) (RR: 3.69, 95% CI: 2.99–4.55, *P* < 0.001) (**Table 1**).

#### 3.4.1 Subgroup analysis

We then conducted a subgroup analysis to investigate the key factors influencing the RR of rash (**Table 1**). Subgroup analyses revealed that the risk of rash was significantly increased according to EGFRIs, trial phase, treatment regimen, and control therapy. In the phase III study, a higher risk of rash was observed. The relative risks of all-grade rash, low-grade rash and high-grade rash were 4.02 (95% CI: 3.38–4.78), 3.87 (95% CI: 2.86–5.24) and 6.74 (95% CI: 4.78–9.49), respectively. All interaction *P* values were less than 0.001. The risk of rash occurrence varies among different types of tumors (interaction *P* values less than 0.05 for all-grade and low-grade rashes). A higher RR was observed for colorectal cancer (RR: 3.32, 95% CI: 2.61–4.23, *P* < 0.001 for all-grade rash, RR: 2.96, 95% CI: 1.76–5.00, *P* < 0.001 for low-grade rash), breast cancer (RR: 3.06, 95% CI: 1.62–5.77, *P* = 0.001 for all-grade rash), and head and neck cancer (RR: 3.58, 95% CI: 2.16–5.91, *P* < 0.001 for low-grade rash). Different treatment regimens had an impact on the relative risk of rash (interaction *P* value less than 0.001). Combination therapy increased the risk of rash, with an RR of 7.98 (95% CI: 6.18–10.31) for all-grade rash, 7.27 (95% CI: 5.34–9.88) for low-grade rash, and 13.70 (95% CI: 9.40–19.97) for high-grade rash. However, regardless of combined use or single use, there was no significant difference in the relative risk of rash among different types of EGFRIs (*P* value greater than 0.05 or RR close to 1). As shown in **Table 1**, regardless of the grade, the relative risk of rash caused by EGFR monoclonal antibodies (EGFR mAbs) is significantly higher than that caused by EGFR-TKIs (*P* < 0.001). More specifically, the RRs of Icotinib, Dacomitinib, Osimertinib and Lazertinib were not significant (*P*> 0.05). Necitumumab (RR: 5.15, 95% CI: 3.05–8.72, *P* < 0.001) and Nimotuzumab (RR: 5.25, 95% CI: 1.23–22.50, *P* = 0.026) may increase the risk of developing all-grade rash, whereas the risk of Aumolertinib (RR: 0.56, 95% CI: 0.42–0.75, *P* < 0.001) was low. Studies published before 2020 reported a greater risk of rash in all-grade rash patients (RR: 2.75, 95% CI: 2.45–3.08, *P* < 0.001) and low-grade rash patients (RR: 2.57, 95% CI: 2.09–3.16, *P* < 0.001). No significant subgroup effects were found for nation (*P* for interaction> 0.05).

#### 3.4.2 Meta-regression analysis

To further explore potential sources of heterogeneity, we performed a meta-regression analysis (**Table 2**). Similar to the subgroup analysis results, phase III studies and combined therapy increased the risk of rash. For the EGFRIs type, the risk of rash increased with the addition of cetuximab, especially for high-grade rashes (RR: 4.01, 95% CI: 1.51–10.63, *P* = 0.006), whereas Aumolertinib significantly reduced the risk of rash (RR: 0.19, 95% CI: 0.04–0.83, *P* = 0.028). Unlike the subgroup analysis results, the regression analysis revealed that the risk of rash was slightly higher in Asians (RR: 1.75, 95% CI: 1.21–2.54, *P* = 0.003), while the type of tumor and publication year had no significant effect on the RR. The reason why results of subgroup analysis and meta-regression were different was that subgroup analysis was univariate and maybe affected by confounding factors. Tumor type and publication year were no longer significant in meta-regression, suggesting that their effects may be explained by other variables (such as treatment plan and drug type). The nation became significant in the meta-regression analysis, suggesting that the country effect was masked by other variables and emerged after adjustment. Therefore, multivariable meta-regression was taken as the primary basis for the results.

**Table 2:** Meta-regression results for the RR of rash.

Variables	All-grade		G1-2		G≥3	
	Exp(b)	*P*- value	Exp(b)	*P*- value	Exp(b)	*P*- value
Nation						
Asian	1.75(1.21-2.54)	0.003	1.84(1.01-1.84)	0.045	1.24(0.68-2.27)	**0.485**
No-Asian	1.24(0.82-1.67)	**0.166**	1.40(0.80-1.40)	**0.233**	1.23(0.77-1.96)	**0.383**
Multinational	ref	–	ref	–	ref	–
Tumor type						
Breast Cancer	0.98(0.49-1.95)	**0.949**	0.83(0.34-0.83)	**0.682**	0.74(0.17-3.17)	**0.68**
Colorectal Cancer	0.96(0.63-1.47)	**0.851**	0.97(0.54-0.97)	**0.917**	0.84(0.40-1.75)	**0.633**
NSCLC	ref	–	ref	–	ref	–
Head and Neck Cancer	0.79(0.49-1.27)	**0.326**	1.31(0.67-1.31)	**0.421**	0.84(0.36-1.94)	**0.675**
Other	0.94(0.58-1.53)	**0.811**	1.09(0.57-1.09)	**0.787**	0.70(0.32-1.53)	**0.37**
Therapeutic regimen						
EGFRI combination vs EGFRI combination	0.30(0.18-0.49)	0.000	0.43(0.20-0.43)	0.031	0.21(0.09-0.47)	0
EGFRI combination vs EGFRI monotherapy	0.34(0.23-0.51)	0.000	0.50(0.26-0.5)	**0.033**	0.33(0.17-0.63)	0.001
EGFRI combination vs other	2.00(1.38-2.91)	0.000	2.48(1.32-2.48)	0.006	2.51(1.30-4.86)	0.007
EGFRI monotherapy vs other	ref	–	ref	–	ref	–
EGFRI						
Afatinib	1.98(1.20-3.27)	0.008	2.68(1.27-2.68)	0.011	1.64(0.67-4.04)	**0.276**
Aumolertinib	0.19(0.04-0.83)	0.028	–	–	–	–
CMAB009	1.78(0.34-9.22)	**0.490**	–	–	1.59(0.09-27.36)	**0.747**
Cetuximab	2.81(1.68-4.72)	0.000	2.42(1.09-2.42)	0.031	4.01(1.51-10.63)	0.006
Dacomitinib	1.18(0.46-2.97)	**0.732**	1.90(0.59-1.90)	**0.276**	2.56(0.56-11.65)	**0.223**
Icotinib	0.39(0.15-1.065)	**0.061**	–	–	1.07(0.16-7.00)	**0.947**
Lazertinib	0.57(0.13-2.59)	**0.465**	–	–	–	–
Osimertinib	1.41(0.60-3.29)	**0.431**	1.21(0.39-1.21)	**0.74**	0.20(0.04-1.13)	**0.068**
Panitumumab	2.29(1.23-4.27)	0.010	3.22(1.16-3.22)	0.025	2.07(0.69-6.27)	**0.194**
Erlotinib	1.58(1.04-2.41)	0.032	1.28(0.62-1.28)	**0.499**	2.00(0.93-4.28)	**0.076**
Gefitinib	ref	–	ref	–	ref	–
Gefitinib or Erlotinib	0.60(0.13-2.79)	**0.511**	0.94(0.19-0.94)	**0.937**	1.86(0.05-67.02)	**0.732**
Nimotuzumab	1.24(0.15-10.14)	**0.841**	–	–	–	–
Necitumumab	1.31(0.54-3.22)	**0.551**	2.24(0.46-2.24)	**0.31**	3.15(0.55-18.15)	**0.197**
AZD8931	4.89(0.19-127.50)	**0.338**	–	–	4.59(0.14-150.38)	**0.39**
Study phase						
Phase II	ref	–	ref	–	ref	–
PhaseIII	1.60(1.21-2.11)	0.001	1.57(1.01-1.57)	0.045	1.65(1.05-2.58)	0.03
Published year						
More earlier	ref	–	ref	–	ref	–
Within 5 years	0.85(0.60-1.21)	**0.365**	0.79(0.46-0.79)	**0.386**	0.84(0.47-1.49)	**0.542**

NSCLC, non-small cell lung cancer; Values with *P* > 0.05 were in bold.

#### 3.4.3 Stratification analysis of therapeutic regimen

Given that the treatment plan is one of the main sources of heterogeneity, we separately compared the impact of different control groups (chemotherapy, targeted therapy, radiotherapy, hormone therapy, etc.) on the incidence and RR of rashes under each treatment plan (**Table 3**). Compared with placebo and chemotherapy, monotherapy with EGFRIs significantly increased the risk of all-grade rashes, while there was no significant difference in the RR among the targeted drugs (different types of EGFRIs drugs, Vandetanib, Trastuzumab, Lapatinib, Duligotuzumab) (*P*> 0.05). The incidence of all grade rashes caused by EGFRIs combination therapy was higher than that caused by all other treatments, but the difference was smaller than that caused by targeted therapy (RR: 3.30, 95% CI: 1.69–9.12, *P*< 0.001) and hormone therapy (RR: 3.13, 95% CI: 1.89–5.20, *P* < 0.001).

**Table 3:** Stratified analysis according to therapeutic regimen.

Groups	All-grade					G1-2					G≥3				
	No. of studies	RR (95% CI)	*P*-value	I^2^ (%)	*P*-interaction	No. of studies	RR (95% CI)	*P*-value	I^2^ (%)	*P*-interaction	No. of studies	RR (95% CI)	*P*-value	I^2^ (%)	*P*-interaction
**EGFRI monotherapy vs other**					<0.001					<0.001					<0.001
Placebo	15	4.12(3.04-5.60)	<0.001	90.60		4	4.23 (2.92-6.13)	<0.001	62.10		14	12.97 (7.53-22.33)	<0.001	10.90	
Chemotherapy	25	6.45 (5.00-8.33)	<0.001	76.10		9	5.54 (3.41-9.02)	<0.001	77.60		25	4.98 (3.41-7.26)	<0.001	6.60	
Targeted therapy	22	0.93 (0.84-1.03)	**0.152**	84.00		9	0.90 (0.81-1.00)	**0.059**	67.50		18	0.99 (0.57-1.71)	**0.956**	78.60	
Chemoradiotherapy	1	0.86 (0.53-1.40)	0.539	-		-	-	-	-		1	-	-	-	
**EGFRI combination vs other**		*Lenalidomide			0.011					**0.43**		*Lenalidomide			**0.218**
Placebo	1	27.00 (1.65-441.43)	0.021	-		1	25.00 (1.52-410.40)	0.024	-		1	3.00 (0.13-71.82)	**0.498**	-	
Chemotherapy	49	8.41 (6.39-11.07)	<0.001	86.40		16	7.80 (5.13-11.89)	<0.001	88.60		47	16.18 (9.76-26.82)	<0.001	60.00	
Targeted therapy	7	3.30 (1.69-9.12)	<0.001	94.40		3	4.02 (0.92-17.64)	**0.065**	93.90		7	5.70 (2.10-15.46)	0.001	56.40	
Chemoradiotherapy	4	9.16(5.86-14.32)	<0.001	13.70		2	10.95 (6.25-19.19)	<0.001	1.30		4	7.96 (3.01-21.03)	<0.001	0.00	
Radiotherapy	3	8.72 (3.31-22.97)	<0.001	41.60		-	-	-	-		2	7.64 (2.68-21.80)	<0.001	21.90	
Hormone Therapy	2	3.13 (1.89-5.20)	<0.001	0.00		-	-	-	-		1	-	-	-	
Chemo-targeted therapy	8	9.86 (3.65-26.59)	<0.001	96.90		2	6.51 (5.13-8.26)	<0.001	0.00		7	27.64 (12.09-63.19)	<0.001	6.80	
Immunotherapy	1	6.94 (3.16-15.22)	<0.001	-		1	5.25 (2.35-11.73)	<0.001	-		1	21.32 (1.28-356.44)	0.033	-	
Other*	1	4.75 (1.95-11.60)	0.001	-		-	-	-	-		1	9.00 (0.52-157.37)	**0.132**	-	
**EGFRI combination vs EGFRI monotherapy**		*Simvastatin			**0.063**		*Simvastatin			**0.416**		*Simvastatin			**0.501**
Chemotherapy	6	1.32 (1.09-1.61)	0.005	1.20		3	1.17 (0.84-1.64)	**0.362**	0.00		6	1.63 (1.13-2.35)	0.009	0.00	
Targeted therapy	23	1.00 (0.96-1.05)	**0.891**	43.70		11	0.90 (0.82-0.99)	0.034	58.80		22	1.28 (0.95-1.73)	**0.106**	57.30	
Immunotherapy	1	1.26 (0.69-2.30)	**0.452**	-		-	-	-	-		1	-	-		
Hormone Therapy	2	1.13 (0.72-1.77)	**0.597**	65.80		1	0.79 (0.54-1.16)	**0.223**	-		2	3.93 (0.71-21.68)	**0.116**	33.50	
Other*	2	0.86 (0.62-1.18)	**0.341**	60.70		1	0.96 (0.74-1.24)	**0.734**	-		2	1.44 (0.24-8.64)	**0.688**	0.00	
**EGFRI combination vs EGFRI combination**		*metformin or motolimod with chemotherapy			0.038		*metformin with chemotherapy			**0.124**		*metformin or motolimod with chemotherapy			**0.672**
Chemotherapy	11	1.02 (0.95-1.10)	**0.550**	32.10		6	1.07 (0.94-1.21)	**0.296**	36.00		11	0.95 (0.65-1.39)	**0.787**	45.40	
Targeted therapy	8	1.13 (0.78-1.64)	**0.528**	76.60		1	0.89 (0.58-1.37)	**0.608**	-		9	1.33 (0.66-2.71)	**0.429**	49.10	
Other*	2	0.73 (0.57-0.95)	0.016	0.00		1	0.75 (0.54-1.05)	**0.09**	-		2	0.84 (0.29-2.48)	**0.754**	0.00	

CI, confidence interval; 13 studies were not estimable for G≥3, of which the events of both groups were 0; Values with *P* > 0.05 were in bold; * for other: other medications used in the therapeutic regimen were explained in each row of stratifications.

#### 3.4.4 Network meta-analysis of EGFRIs monotherapy study

**Figure 2:**
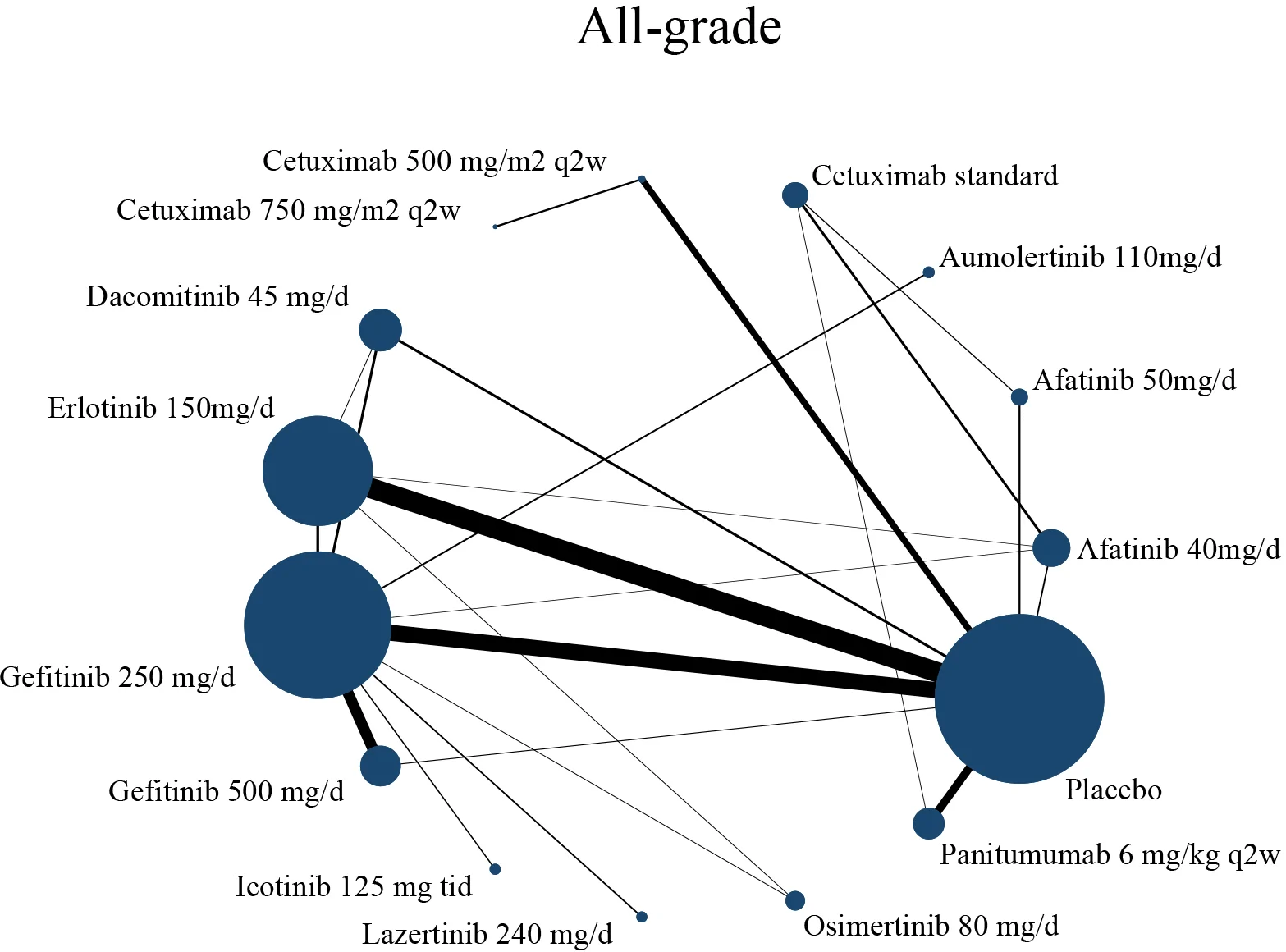
Network diagrams of the network meta-analysis for all-grade rash. The size of the nodes corresponds to the number of patients randomly assigned to that intervention. Interventions directly compared are connected by a line; the thickness of the line represents the number of trials evaluating that comparison.

To compare the effects of EGFRIs types and dose directly on the RR of rash, we conducted a network meta-analysis of studies that compared EGFRI monotherapy with placebo or other single EGFRI to ensure that the heterogeneity was not significant, thereby ensuring transferability. All included 33 studies with 15,739 patients were shown in **Supplementary Table 6**. The network geometric diagram of the results was shown in **Figure 2**. Although the test of global inconsistency revealed no significant difference (*P* = 0.1079), local inconsistency was found in node and loop inconsistency (**Tables 4**, **5**). Results showed that the direct and indirect evidence effect values of Gefitinib 250 mg/d, Gefitinib 500 mg/d and placebo, as well as Afatinib, Cetuximab, Panitumumab and placebo were inconsistent. Thus, indirect comparisons involving these parts were not completely reliable.

**Table 4:** Node-splitting analysis of inconsistency.

Nodes	Direct		Indirect		Difference		*P>z*
	Coef.	Std. err.	Coef.	Std. err.	Coef.	Std. err.	
Afatinib 40mg/d, Cetuximab standard	0.251314	0.426611	0.726877	0.5245595	-0.4755627	0.676136	0.482
Afatinib 40mg/d, Erlotinib 150mg/d	0.01128	0.369094	0.08272	0.2883943	-0.0714396	0.468404	0.879
Afatinib 40mg/d, Gefitinib 250 mg/d	-0.01672	0.36586	-0.28805	0.2814207	0.2713278	0.461575	0.557
Afatinib 40mg/d, Placebo	-1.31811	0.389958	-1.34362	0.2704115	0.0255139	0.474541	0.957
Afatinib 50mg/d, Cetuximab standard	-0.02603	0.376826	0.311984	0.5885986	-0.3380144	0.698889	0.629
Afatinib 50mg/d, Placebo	-1.59318	0.400412	-1.93119	0.5728135	0.3380124	0.698888	0.629
Aumolertinib 110 mg/d, Gefitinib 250 mg/d	0.571951	0.381777	-0.94174	634.8635	1.513695	634.8637	0.998
Cetuximab standard, Panitumumab 6 mg/kg q2w	0.017609	0.346711	1.612892	0.8657262	-1.595283	0.932572	0.087
Cetuximab 500 mg/m2 q2w, Cetuximab 750 mg/m2 q2w	0.132172	0.390689	-2.97757	644.3196	3.109743	644.3197	0.996
Cetuximab 500 mg/m2 q2w, Placebo	-2.88538	0.669598	-1.02111	282.9458	-1.864274	282.9469	0.995
Dacomitinib 45 mg/d, Erlotinib 150mg/d	-0.06766	0.362645	-0.45633	0.3324112	0.3886689	0.491944	0.429
Dacomitinib 45 mg/d, Gefitinib 250 mg/d	-0.44217	0.431077	-0.56481	0.3130692	0.1226418	0.532766	0.818
Dacomitinib 45 mg/d, Placebo	-2.03535	0.416379	-1.47178	0.3022994	-0.563567	0.514545	0.273
Erlotinib 150mg/d, Gefitinib 250 mg/d	-0.12399	0.212287	-0.38123	0.2289749	0.2572419	0.312241	0.41
Erlotinib 150mg/d, Osimertinib 80 mg/d	-0.30469	0.365519	-0.88306	0.7213116	0.5783769	0.807878	0.474
Erlotinib 150mg/d, Placebo	-1.45596	0.221006	-1.32044	0.2285779	-0.135527	0.317255	0.669
**Gefitinib 250 mg/d, Gefitinib 500 mg/d**	0.348181	0.122566	-1.75056	0.4758715	2.098739	0.492511	**0**
Gefitinib 250 mg/d, Icotinib 125 mg tid	-0.19553	0.369279	0.1839	631.8646	-0.3794311	631.8647	1
Gefitinib 250 mg/d, Lazertinib 240 mg/d	-0.0089	0.376347	0.339605	639.6467	-0.3485021	639.6468	1
Gefitinib 250 mg/d, Osimertinib 80 mg/d	-0.29746	0.365591	0.280912	0.7212032	-0.5783771	0.807878	0.474
Gefitinib 250 mg/d, Placebo	-1.05692	0.198439	-1.26476	0.2274737	0.2078435	0.300968	0.49
**Gefitinib 500 mg/d, Placebo**	-0.59409	0.264535	-1.7895	0.2043373	1.195401	0.334841	**0**
Panitumumab 6 mg/kg q2w, Placebo	-3.15709	0.793349	-1.5618	0.4901936	-1.595296	0.932574	0.087

Values with *P* < 0.05 and inconsistent nodes were in bold.

**Table 5:** Loop analysis of inconsistency.

Loop	ROR	*z-*value	*p-*value	95%CI
**Afa50-Cetsta-Pan6-Placebo**	4.818	2.112	**0.035**	(1.12,20.73)
**Afa40-Cetsta-Pan6-Placebo**	4.807	2.051	**0.04**	(1.07,21.56)
Gef250-Gef500-Placebo	2.482	1.569	0.117	(1.00,7.73)
Dac45-Gef250-Placebo	1.685	0.521	0.602	(1.00,12.01)
Dac45-Erl150-Placebo	1.635	0.604	0.546	(1.00,8.05)
Erl150-Gef250-Plcebo	1.318	0.694	0.487	(1.00,2.87)
Dac45-Erl150-Gef250	1.283	0.828	0.407	(1.00,2.32)
Afa40-Gef250-Placebo	1.259	0.24	0.81	(1.00,8.23)
Erl150-Gef250-Osi80	1.222	1.938	0.053	(1.00,1.50)
Afa40-Erl150-Placebo	1.158	0.186	0.853	(1.00,5.45)
Afa40-Erl150-Gef250	1.102	0.504	0.614	(1.00,1.61)
Afa40-Afa50-Cetsta-Placebo	1.002	0.007	0.994	(1.00,1.90)

CI, confidence interval; Values with *P* < 0.05 and texts of inconsistent loops were in bold.

Considering the influence of small sample size and single evidence, we conducted a sensitivity analysis. After removing five studies, the inconsistency disappeared (**Supplementary Tables 7**, **8**), suggesting that the overall network inconsistency might stem from a few small sample size and anomalous studies. However, due to the deletion, the network geometric diagram was sparser than before (**Supplementary Figure 4**), making the robustness of the indirect path was limited.

**Figure 3:**
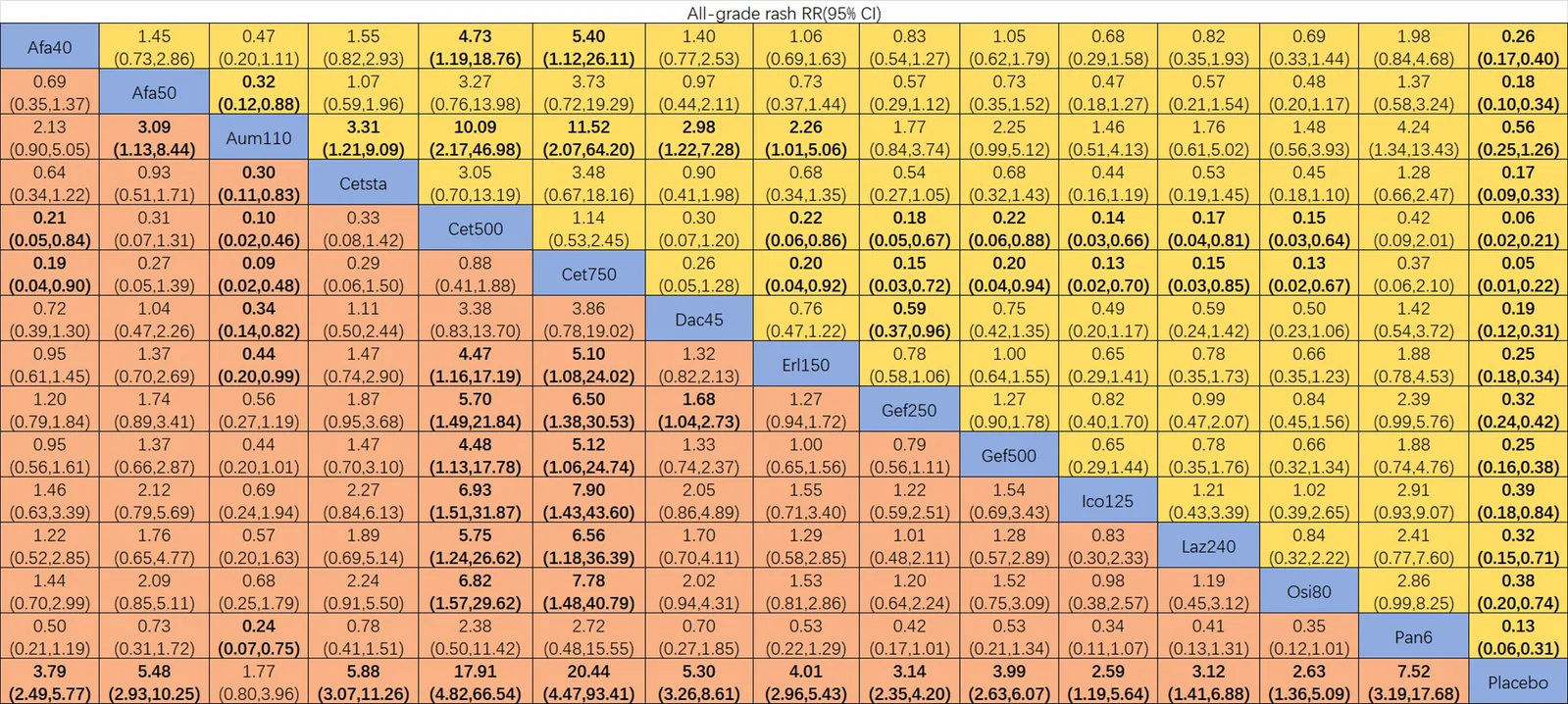
Pooled estimates of the network meta-analysis. The data in each cell represent risk ratios (95% confidence intervals) when the treatment defined in the column is compared with the treatment defined in the row is compared; a value greater (smaller) than 1 indicates that the column-defining treatment is more (less) toxic than the row-defining treatment is. Significant results are indicated in bold.

**Figure 4:**
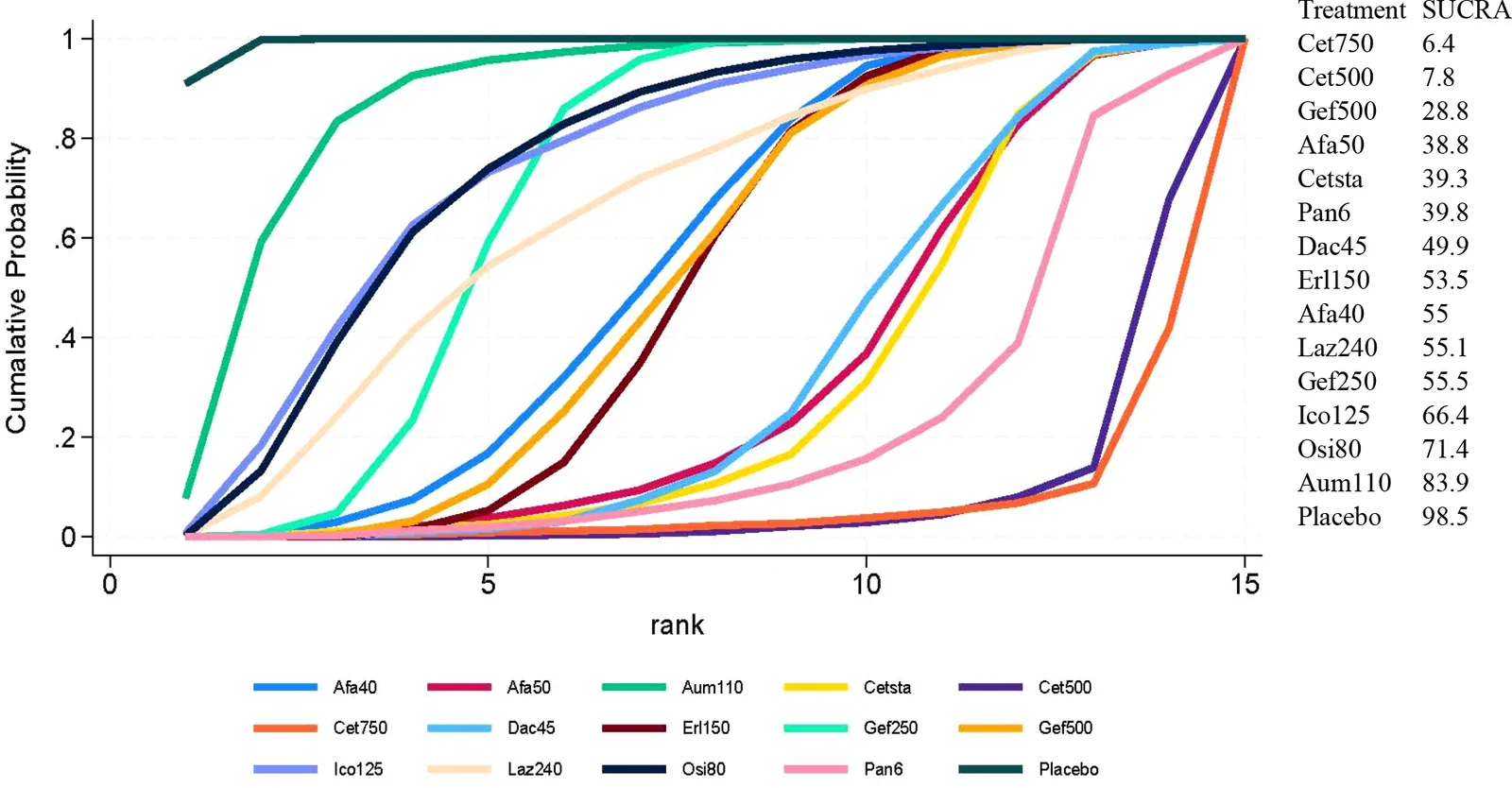
Relative rankings of treatments. The earlier the curve approaches 1, the greater the safety of the intervention measure. Afa40=Afatinib 40 mg/d, Afa50=Afatinib 50 mg/d, Aum110=Aumolertinib 110 mg/d, Cetsta=Cetuximab standard (400 mg/m2 on day 1 followed 250 mg/m2 weekly), Cet500=Cetuximab 500 mg/m2 q2w, Cet750=Cetuximab 750 mg/m2 q2w, Dac45=Dacomitinib 45 mg/d, Erl150=Erlotinib 150 mg/d, Gef250=Gefitinib 250 mg/d, Gef500= Gefitinib 500 mg/d, Ico125=Icotinib 125 mg tid, Laz240=Lazertinib 240 mg/d, Osi80=Osimertinib 80 mg/d, Pan6=Panitumumab 6 mg/kg q2w.

According to the results shown in **Figure 3**, **4**, Aumolertinib (SUCRA = 83.9), Osimertinib (SUCRA = 71.4), and Icotinib (SUCRA = 71.4) ranked the top three. Compared with Cetuximab (RR: 0.30, 95% CI: 0.11–0.83), Dacomitinib (RR: 0.34, 95% CI: 0.14–0.82), Erlotinib (RR: 0.44, 95% CI: 0.20–0.99), and Panitumumab (RR: 0.24, 95% CI: 0.07–0.75), Aumolertinib had a relative low RR. While high-dose Cetuximab had a greater risk of rash than Afatinib, Aumolertinib, Erlotinib, Gefitinib, Icotinib, Lazertinib, and Osimertinib. We also conducted SUCRA ranking and pooled estimates for the remaining studies after sensitivity analysis. Except for the changes in drug rankings and RR significance about drugs involving local inconsistencies, the main results were consistent (**Supplementary Figure 5**, **6**).

### 3.5 Heterogeneity and sensitivity analysis

There was significant heterogeneity among the different studies (*I^2^*>50%). Meta-regression explained 58.78% of the observed heterogeneity (R²) in all-grade rashes, 70.78% in low-grade rashes and 57.24% in high-grade rashes, indicating that more than half of the variability was accounted for by the included moderators (**Table 6**). The reason why there is still considerable heterogeneity may be that the number of zero events in the control group is relatively large. Continuity correction leads to a systematic underestimation of RR, resulting in an increase in *I^2^*.

**Table 6:** Test for meta-regression.

	All-grade	G1-2	G≥3
*τ²*	0.49	0.35	0.72
*I² (%)*	92.36	87.83	59.88
*R^2^(%)*	58.78	70.78	57.24
F (*P*)	9.67 (*P*<0.001)	7.06 (*P*<0.001)	6.50 (*P*<0.001)

The restricted maximum likelihood (REML) estimation method was used to estimate the residual between-study variance (τ²=0.49 in all-grade rash, τ²=0.35 in low-grade rash, τ²=0.72 in high-grade rash). The degree of heterogeneity across studies was substantial, as indicated by the high I² >50%, suggesting that the variability in effect sizes was due to real differences between studies rather than chance. The overall test of moderators was statistically significant (P<0.001), indicating that the included predictors significantly explain the variation in effect sizes across studies. The restricted maximum likelihood (REML) estimation method was used to estimate the residual between-study variance (τ²=0.49 for all-grade rash, τ²=0.35 for low-grade rash, and τ²=0.72 for high-grade rash). The degree of heterogeneity across studies was substantial, as indicated by the high *I²* >50%, suggesting that the variability in effect sizes was due to real differences between studies rather than chance. The overall test of moderators was statistically significant (*P* < 0.001), indicating that the included predictors significantly explain the variation in effect sizes across studies.

**Figure 5:**
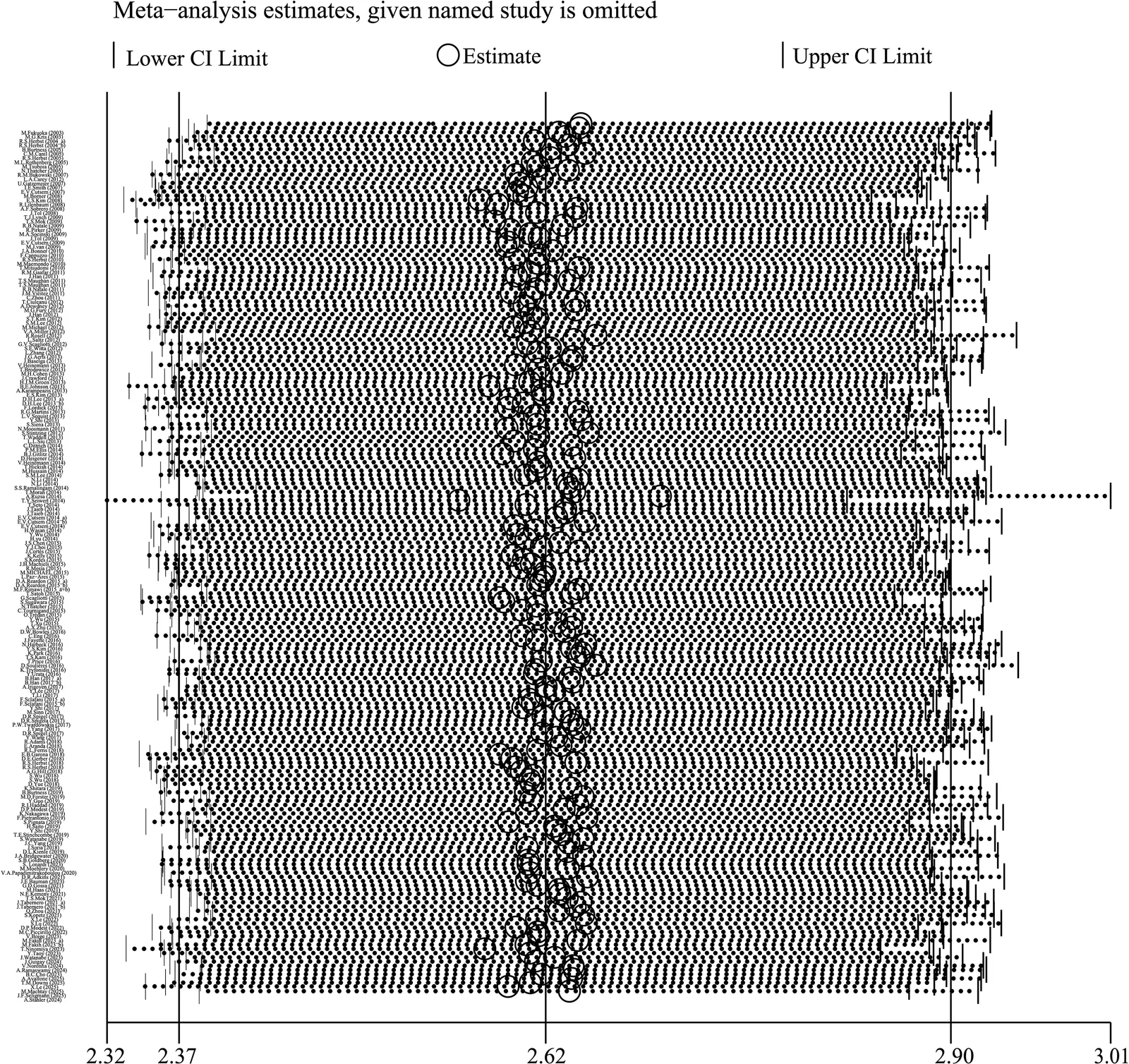
Sensitivity analysis.

Sensitivity analyses (**Figure 5**) revealed no significant change after omitting each study reporting all-grade rash or low-grade or high-grade rash in succession (including studies at high risk of bias and unclear bias), suggesting that the results of our study were robust.

### 3.6 Publication bias assessment

**Figure 6:**
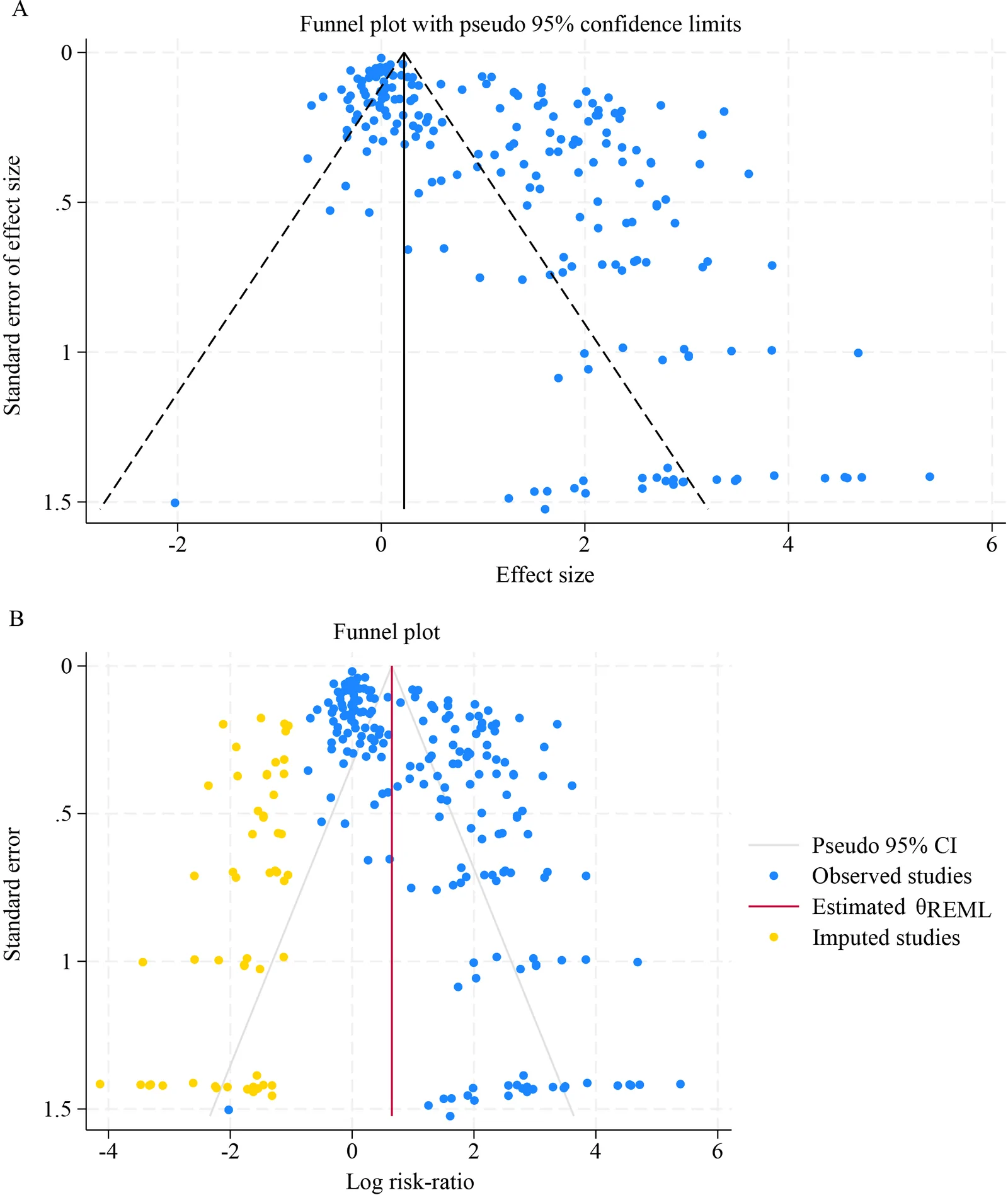
**(A)** Funnel plot of data from the meta-analysis. **(B)** trim-and-fill adjusted funnel plot of data from the meta-analysis.

With respect to the RR of rash, publication bias was detected by funnel plots (**Figure 6A**) and the Egger test (*P* < 0.001), which indicate that risks may be affected by small-study effect. We therefore adopted the trim-and-fill method to evaluate the number of deficient studies (**Figure 6B**). After supply 54 virtual missing data, the RR decreased from 3.04 (95% CI: 2.58-3.59) to 1.92 (95% CI: 1.59–2.31). However, the RR remains greater than 1, indicating that the results are reliable although the actual level of risk is likely to be more conservative. Combining sensitivity analysis and trimming methods did not alter the magnitude of the effect size. Therefore, we think that the results are not subject to publication bias.

## 4 Discussion

In this systematic review and meta-analysis, we summarized 194 RCT studies (involving a total of 65,116 participants) and provided a relatively comprehensive overview of the incidence and relative risk of rash associated with different EGFRIs. The combined incidence of all-grade rashes in patients receiving EGFRIs treatment was 52.30%, and the combined incidence of high-grade (≥3 grade) rashes was 9.50%. Compared with the control, EGFRIs treatment significantly increased the risk of all-grade rashes (RR = 2.62) and high-grade rashes (RR = 3.69). Whereas overall estimate is not directly applicable to any single EGFRIs agent or regimen, due to the high heterogeneity of the pooled incidence.

To the best of our knowledge, our study is the largest meta-analysis evaluating the risk of rash associated with EGFRIs, including updated studies with larger sample sizes, broader drug coverage, and applying network meta-analysis. Similar to the results of Wenxia Sun (27) and Xi Zhang (28), we found that the type of EGFR-TKI is an important influencing factor for the risk of rash occurrence, but newly added third-generation EGFR-TKIs, whose risk of developing rash was significantly lower than the two above. Due to the inclusion of new data (15, 29), the incidence of rashes for some drugs has changed. For instance, the RR of the rash in dacomitinib and necitumumab was higher than that in previous studies (30), while Cetuximab (RR = 3.88) and Panitumumab (RR = 3.50) showed no significant difference.

We additionally explored the potential risk factors for EGFRIs-associated rash and reported that the RR of rash may be associated with ethnicity. Our regression analysis suggested that Asian individuals may have higher risk than other individuals to develop rash. Some studies have reported that the incidence of adverse skin reactions in the Asian population is slightly higher than that in the general population (31–33) or that drug dosages need to be adjusted to continue treatment (34, 35). This might be because the stratum corneum of Asians is thinner, making them more sensitive to stimuli and more prone to overreaction (19). In addition, activating EGFR gene mutations is most common in Asians, leading to an increased response to EGFR TKIs (36).

The risk of EGFRI rash in the phase III trial was significantly greater than that in the phase II trial. The reason might be that the sample size increased significantly (with 14067 participants in the phase II study and 50131 in the phase III study), and the duration of treatment was prolonged (ranging from 3.25–64.00 months *vs* 3.30–88.10 months).

The type of EGFRIs is one of the important factors influencing the RR of rashes. The incidence of rash associated with third-generation EGFR-TKIs, especially Aumolertinib, is relatively low. This might be because third-generation EGFR-TKIs are pyrimidine-based drugs and have higher selectivity than first- and second-generation quinazolinine-based drugs do (37, 38). The structure of Aumolertinib is based on Osimertinib by removing a cyclopropyl group on the indole nitrogen, which inhibits the generation of nonselective metabolites that inhibit wild-type EGFR (37). Unlike EGFR-TKIs, which inhibit intracellular tyrosine kinases, EGFR mAbs bind to the extracellular domain of EGFR to inhibit receptor function (39) and simultaneously activate immune-mediated cytotoxic responses to exert anticancer effects (40, 41). This may be the reason why the risk of rash occurrence in patients receiving EGFR mAbs is relatively high. Although panitumumab and cetuximab have different chemical structures, administration methods, and receptor affinities, no significant differences in rashes have been reported in other head-to-head studies (42, 43). Necitumumab binds to the receptor to the maximum extent and patients with smaller body surface areas have a greater exposure to Necitumumab compared to Cetuximab (41). This may affect the comparison between Necitumumab and Cetuximab. Combination therapy, especially when combined with radiotherapy or chemotherapy, indicate an increased risk of skin toxicity. This might be because when EGFRIs are used in combination with chemotherapy drugs or radiotherapy, a synergistic effect occurs, which enhances their toxicity and can lead to general immunity and infection, causing the rash to worsen and appear earlier (44). Additionally, another study revealed that transforming growth factor-β1, a negative immunomodulator and a key molecule that mediates the response of normal tissues to radiation, might increase the skin toxicity of the combination of cetuximab and radiotherapy (45).

In addition to being highly expressed in tumor cells, EGFR is highly expressed in normal skin structures such as keratinocytes, hair follicles, and sebaceous glands and plays crucial roles in their development and physiology. It can promote the proliferation and differentiation of keratinocytes and inhibit the production of chemokines. The detailed mechanism of EGFRIs-induced rash is not yet clear, and there is evidence indicating that EGFRIs affect the growth and migration of keratinocytes, increase apoptosis and adhesion, causing damage to the stratum corneum, hair follicle blockage, and the inhibition of antimicrobial peptide production via the inhibition of the EGFR/PI3K/AKT/mTOR or EGFR/Ras/Raf/MAPK/ERK pathways. Moreover, it stimulates proinflammatory cytokines such as CC-chemokine ligand 2, CC-chemokine ligand 5, interleukin (IL)-36γ and IL-8 and recruits inflammatory cells, leading to disruption of the skin barrier; infiltration of inflammatory cells; and clinical manifestations, including mainly erythema, papules and dryness (46). During EGFRIs treatment, the reduction in the expression of antibacterial molecules such as antimicrobial peptides and the imbalance of the skin microbiota are closely related to the development of rash, which may even lead to secondary infections (47, 48). Furthermore, polymorphisms in the EGFR gene and related antigens are also associated with the occurrence of acneiform rash to some extent (49, 50).

Our research data quantified risks, so it might provide a reference basis for doctors to fully inform patients before initiating treatment, manage their expectations, and improve compliance. For Asian patients using EGFR mAbs, in combination with radiotherapy and chemotherapy, doctors might consider strengthening preventive measures earlier (such as early use of moisturizers and sunscreens, attention to skin care, and topical hormone application), and closely monitoring the occurrence of related adverse reactions. Moreover, the rash risk spectrum can be taken into account as one of the factors for drug selection when a treatment plan is formulated, especially when the therapeutic effects are comparable.

Although we have made every effort to minimize the influence of confounding variables, our analysis still presents certain limitations. First, this meta-analysis was based on published studies rather than on individual patient data, and potential confounding factors, such as age, gender, and specific dosage, could not be adjusted. Despite our efforts to minimize the effects of heterogeneity through subgroup analysis and random effects models, there are still unmeasured factors, including varying follow-up durations, a wide time span, differing sample sizes, and tumor staging etc. and may exacerbate heterogeneity. Consequently, the robustness of the results is somewhat compromised, and clinical recommendations remain exploratory. Second, although the inconsistency disappeared after the sensitivity analysis in the network meta-analysis, the transitivity is only partially satisfied, unmeasured confounding across eras and tumor types may bias the results. The robustness of indirect evidence was limited, indicating the SUCRA ranking can only be used as a reference of a greater possibility. Final, some studies may not have fully reported all levels of adverse reactions, and studies with negative results and small samples may lead to possible deviations in the results. Given the limitations of this study, future research can collect individual patient data for more precise analysis to identify risk factors. More prospective studies are needed to evaluate the effectiveness of preventive intervention measures in reducing the incidence and severity of rashes.

## 5 Conclusions

In conclusion, our analysis indicated that the use of EGFRIs significantly increases the risk of rashes. In addition, subgroup analysis and regression analysis showed that the risk of rash varied according to race, different EGFRIs types, trial stage, and different treatment regimens but was not significantly correlated with tumor type or publication year. Factors that increase the risk of rash occurrence include the Asian population, EGFR monoclonal antibodies, phase III clinical trials, and EGFRIs combined with chemoradiotherapy, whereas Aumolertinib is associated with a low risk of developing rash. These findings may help doctors recognize the risk of rash associated with EGFRIs and may provide reference in adjusting dosages and plans to suit individual patients. Moreover, on the basis of the current research results on the pathophysiological mechanism of rashes, we believe that the barrier defects and abnormal inflammation induced by EGFRIs are the precise pathological basis of the clinical phenotype of rashes. However, further research is still needed to clarify the pathogenesis of this rash.

## Data Availability

The original contributions presented in the study are included in the article/**Supplementary Material**. Further inquiries can be directed to the corresponding author.

## Supplementary material

Supplementary Figure 1 Assessment of risk bias traffic light plots and summary plots. **(A)** Traffic light plot. **(B)** Summary plot.

Supplementary Figure 2 Forest plot of the analysis of the incidence of rash.

Supplementary Figure 3 Forest plot of the analysis for the relative risk of rash.

Supplementary Figure 4 Network diagrams of the network meta-analysis for all-grade rash after removing five small sample size and anomalous studies.

Supplementary Figure 5 Relative rankings of treatments after removing five small sample size and anomalous studies.

Supplementary Figure 6 Pooled estimates of the network meta-analysis after removing five small sample size and anomalous studies.

SUPPLEMENTARY TABLE 1 Search strategies for PubMed.

SUPPLEMENTARY TABLE 2 Search Strategies for Embase.

SUPPLEMENTARY TABLE 3 Search Strategies for Cochrane.

SUPPLEMENTARY TABLE 4 Search Strategies for Web of Science.

SUPPLEMENTARY TABLE 5 Baseline characteristics of the included studies.

SUPPLEMENTARY TABLE 6 The baseline characteristics of the studies included in network-meta.

SUPPLEMENTARY TABLE 7 Node-splitting analysis of inconsistency after sensitivity analysis.

SUPPLEMENTARY TABLE 8 Loop analysis of inconsistency after sensitivity analysis.
