# Supplementary material for: Rash associated with epidermal growth factor receptor inhibitors: a systematic review and meta-analysis of 194 randomized controlled trials
Source: Front Immunol. 2026 Aug 21;17:1814337. doi: 10.3389/fimmu.2026.1814337 (PMC13541753; doi:10.3389/fimmu.2026.1814337)
Supplement: Supplementary file 1 [file DataSheet1.pdf]

A

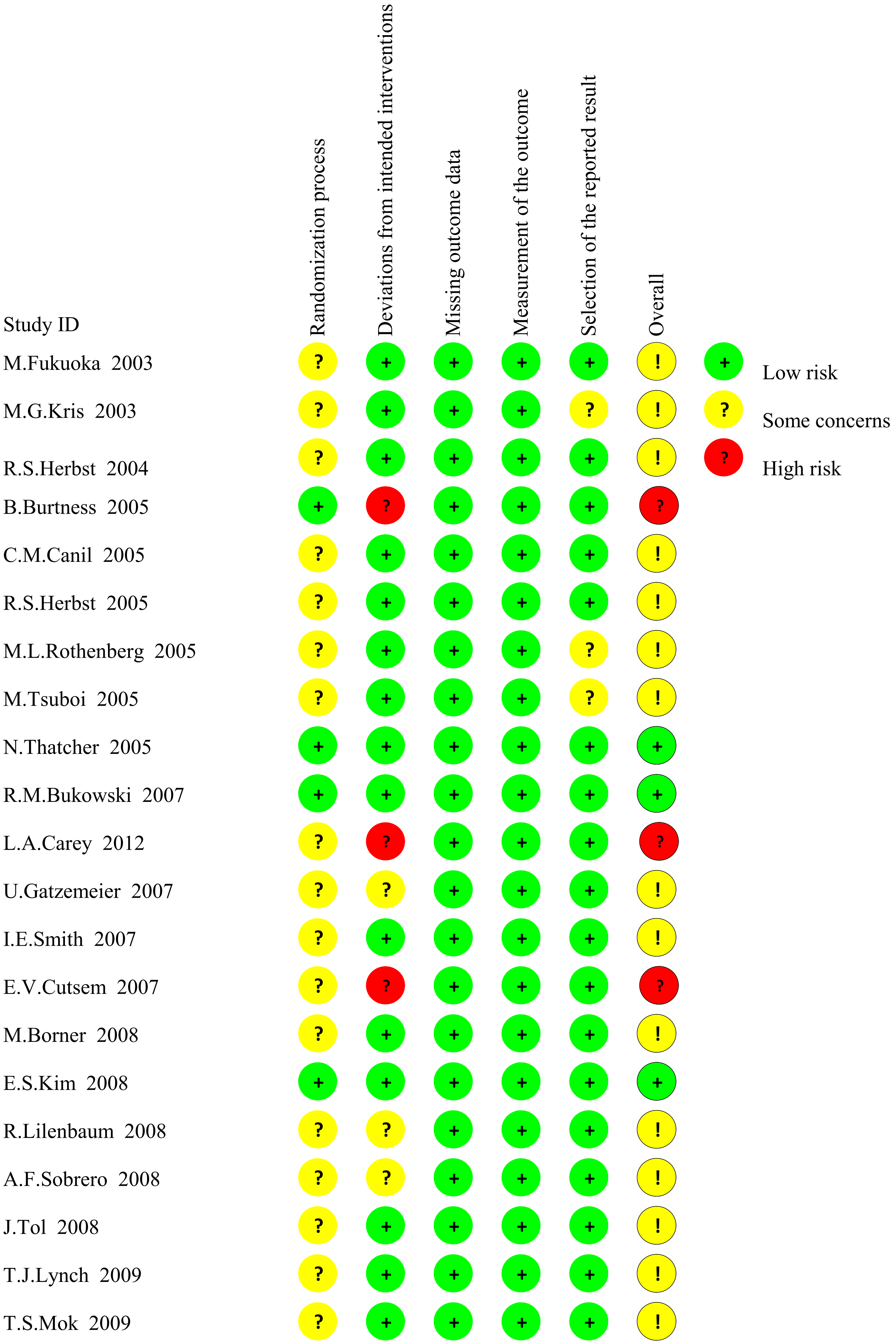
Study ID
Randomization process
Deviations from intended interventions
Missing outcome data
Measurement of the outcome
Selection of the reported result
Overall
M.Fukuoka 2003
M.G.Kris 2003
R.S.Herbst 2004
B.Burtness 2005
C.M.Canil 2005
R.S.Herbst 2005
M.L.Rothenberg 2005
M.Tsuboi 2005
N.Thatcher 2005
R.M.Bukowski 2007
L.A.Carey 2012
U.Gatzemeier 2007
I.E.Smith 2007
E.V.Cutsem 2007
M.Borner 2008
E.S.Kim 2008
R.Lilenbaum 2008
A.F.Sobrero 2008
J.Tol 2008
T.J.Lynch 2009
T.S.Mok 2009
Low risk
Some concerns
High risk

R.B.Natale 2009	?	+	+	+	+	!
R.Pirker 2009	+	?	+	+	+	!
M.A.Socinski 2009	?	+	+	+	+	!
J.Tol 2009	?	?	+	+	+	!
E.V.Cutsem 2009	?	?	+	+	+	!
M.J.van 2009	?	+	+	+	+	!
J.A.Bonner 2010	+	+	+	+	+	+
F.Cappuzzo 2010	+	+	+	+	+	+
R.S.Herbst 2010	?	+	+	+	+	!
M.Maemondo 2010	?	+	+	+	+	!
T.Mitsudomi 2010	+	?	+	+	+	!
R.M.Gaafar 2011	?	+	+	+	+	!
J.Han 2011	?	+	+	+	+	!
T.S.Maughan 2011	+	+	+	+	+	+
R.B.Natale 2011	?	+	+	+	+	!
J.M.Viéitez 2011	?	+	+	+	+	!
C.Zhou 2011	+	?	+	+	+	!
T.Ciuleanu 2012	+	+	+	+	+	+
A.Dewdney 2012	?	+	+	+	+	!
M.G.Fury 2012	?	+	+	+	+	!
J.Han 2012	?	?	+	+	+	!
S.T.Kim 2012	+	+	+	+	+	+
S.M.Lee 2012	+	+	+	+	+	+
M.Michael 2012	?	+	+	+	+	!
V.A.Miller 2012	+	+	+	+	+	+
R.Rosell 2012	+	+	+	+	+	+
L.Saltz 2012	+	?	+	+	+	!
G.V.Scagliotti 2012	+	+	+	+	+	+
S.E.Witta 2012	?	+	+	+	+	!

L.Zhang 2012	+	+	+	+	+	+
J.G.Aerts 2013	?	+	+	+	+	!
J.Baselga 2013	+	+	+	+	+	+
V.Heinemann 2013	+	+	+	+	+	+
T.Brodowicz 2013	?	+	+	+	+	!
M.H.Cohen 2013	+	?	+	+	+	!
J.Crawford 2013	?	+	+	+	+	!
H.J.M.Groen 2013	+	+	+	+	+	+
B.E.Johnson 2013	+	+	+	+	+	+
A.Karampeazis 2013	+	+	+	+	+	+
E.S.Kim 2013	+	+	+	+	+	+
D.H.Lee 2013	+	?	+	+	+	!
F.Lordick 2013	+	+	+	+	+	+
R.G.Martins 2013	+	+	+	+	+	+
L.V.Sequist 2013	?	+	+	+	+	!
Y.Shi 2013	+	+	+	+	+	+
S.Siena 2013	?	?	+	+	+	!
N.Moosmann 2011	+	+	+	+	+	+
S.Stintzing 2012	?	?	+	+	+	!
T.Waddell 2013	+	+	+	+	+	+
L.L.Siu 2013	+	+	+	+	+	+
C.Dittrich 2014	?	?	+	+	+	!
P.M.Ellis 2014	+	+	+	+	+	+
B.J.Gitlitz 2014	?	+	+	+	+	!
D.Heigener 2014	?	?	+	+	+	!
V.Heinemann 2014	+	+	+	+	+	+
T.Hickish 2014	?	+	+	+	+	!
M.Hussain 2014	?	+	+	+	+	!
S.M.Lee 2014	+	+	+	+	+	+

N.Li 2014	?	+	+	+	+	!
N.Li 2014	?	+	+	+	+	!
S.S.Ramalingam 2014	+	+	+	+	+	+
T.Moran 2014	?	+	+	+	+	!
A.Ruzsa 2014	?	+	+	+	+	!
T.Y.Seiwert 2014	?	+	+	+	+	!
T.Seto 2014	+	+	+	+	+	+
J.Taieb 2014	+	+	+	+	+	+
E.V.Cutsem 2014	?	+	+	+	+	!
E.V.Cutsem 2014	+	+	+	+	+	+
H.Wasan 2014	?	+	+	+	+	!
Y.Wu 2014	+	+	+	+	+	+
H.yu 2014	?	+	+	+	+	!
J.S.Chen 2015	+	+	+	+	+	+
Y.J.Choi 2015	?	+	+	+	+	!
J.Cortés 2015	+	+	+	+	+	+
K.Kelly 2015	?	+	+	+	+	!
S.Kordes 2015	+	+	+	+	+	+
J.H.Machiels 2015	+	+	+	+	+	+
R.Mesía 2015	+	+	+	+	+	+
M.MICHAEL 2015	?	?	+	+	+	!
L.Paz-Ares 2015	+	+	+	+	+	+
D.A.Reardon 2015	?	+	+	+	+	!
M.F.Rimawi 2015	+	+	+	+	+	+
T.Satoh 2015	?	+	+	+	+	!
G.Scagliotti 2015	?	+	+	+	+	!
S.Sugawara 2015	?	+	+	?	+	!
N.Thatcher 2015	+	+	+	+	+	+
C.Tournigand 2015	?	+	+	+	+	!

O.Trédan 2015	?	+	+	+	+	!
Y.Wu 2015	?	+	+	+	+	!
T.Xu 2015	?	?	+	+	+	!
A.X.Zhu 2015	+	+	+	+	+	+
D.W.Bowles 2016	?	?	+	+	+	!
C.Eng 2016	+	?	+	+	+	!
J.Fayette 2016	+	+	+	+	+	+
N.Harbeck 2016	+	+	+	+	+	+
Y.S.Kim 2016	?	+	+	+	+	!
K.Park 2016	+	+	+	+	+	+
T.S.Kam 2016	?	+	+	+	+	!
T.Price 2016	?	?	+	+	+	!
D.Soulières 2016	+	?	+	+	+	!
K.Tryfonidis 2016	?	+	+	+	+	!
Y.Urata 2016	+	+	+	+	+	+
B.Han 2017	?	+	+	+	?	!
A.Irigoyen 2017	?	+	+	+	+	!
Y.Lee 2017	?	+	+	+	+	!
T.Li 2017	?	+	+	+	+	!
F.Sclafani 2015	?	+	+	+	+	!
Y.Shi 2017	+	?	+	+	+	!
M.Sinn 2017	+	+	+	?	+	!
D.R.Spigel 2017	?	+	+	+	+	!
D.R.Spigela 2017	?	?	+	?	+	!
P.W.Twardowskia 2017	?	+	+	+	+	!
Y.Wang 2017	?	+	+	+	+	!
J.Yang 2017	+	?	+	+	+	!
D.R.Spigel 2017	?	+	+	+	+	!
R.Adams 2018	+	+	+	+	+	+

E.Aranda 2018	?	+	+	+	+	!
R.L.Ferris 2018	?	+	+	+	+	!
E.B.Garona 2018	+	?	+	?	+	!
D.E.Gerber 2018	?	+	+	+	+	!
R.S.Herbst 2018	+	+	+	+	+	+
A.G.Hill 2018	?	+	+	+	+	!
S.Wu 2018	?	+	+	+	+	!
D.Yue 2018	+	+	+	+	+	+
K.Shitara 2019	?	+	+	+	+	!
B.Burtness 2019	+	+	+	+	+	+
M.D.Forster 2019	?	+	+	+	+	!
D.P.Modest 2019	+	+	+	+	+	+
Y.Guo 2019	+	+	+	+	+	+
R.I.Haddad 2019	?	+	+	?	+	!
K.Nakagawa 2019	+	+	+	+	+	+
F.Pietrantonio 2019	?	+	+	+	+	!
S.Pignata 2019	+	+	+	+	+	+
H.Saito 2019	+	+	+	+	+	+
Y.Shi 2019	?	?	+	+	+	!
T.E.Stinchcombe 2019	?	+	+	+	+	!
S.Watanabe 2019	?	?	+	?	+	!
J.C.Yang 2019	?	+	+	+	+	!
J.Soria 2018	?	+	+	+	+	!
D.L.Kienle 2019	?	?	+	+	+	!
S. Lonardi 2023	?	?	+	+	+	!
J.A.Bridgewater 2020	+	+	+	+	+	+
S.B.Goldberg 2020	?	+	+	+	+	!
M.Moehlery 2020	?	?	+	?	+	!
V.A.Papadimitrakopoulou 2020	?	+	+	+	+	!

J.E Bauman 2023	?	?	+	+	+	!
D.R.Adkins 2021	?	+	+	+	+	!
G.D.Gossa 2021	?	+	+	+	+	!
T.S.Mok 2021	?	?	+	+	+	!
M.Haas 2021	?	+	+	+	+	!
N.E.Kemeny 2021	?	?	+	+	+	!
J.Tabernero 2021	?	?	+	+	+	!
Q.Zhou 2021	?	+	+	+	+	!
S.Kopetz 2021	?	+	+	?	+	!
S.Lu 2022	?	+	+	+	+	!
X.Le 2022	+	+	+	?	+	!
D.P.Modest 2022	+	+	+	+	+	+
M.C.Piccirillo 2022	?	+	+	+	+	!
V.Boige 2023	?	+	+	+	+	!
M.Fakih 2023	?	+	+	+	+	!
T.Ninomiya 2023	?	+	+	+	+	!
Y.Taoy 2023	?	+	+	+	+	!
J.Watanabe 2023	?	+	+	+	+	!
J.Guigay 2024	?	+	+	+	+	!
B.C.Cho 2023	?	+	+	+	+	!
V.Noronha 2024	?	?	+	+	+	?
A.Ramaswamy 2024	?	+	+	+	+	!
A.Stahler 2024	+	?	+	+	+	!
J.F.Seligmann 2025	+	+	+	+	?	!
M.Machtay 2025	+	+	+	+	+	+
X.Le 2025	+	+	+	+	+	+
T.M.Downs 2025	+	+	+	+	?	!
A.Avallone 2025	+	+	+	+	+	+

B

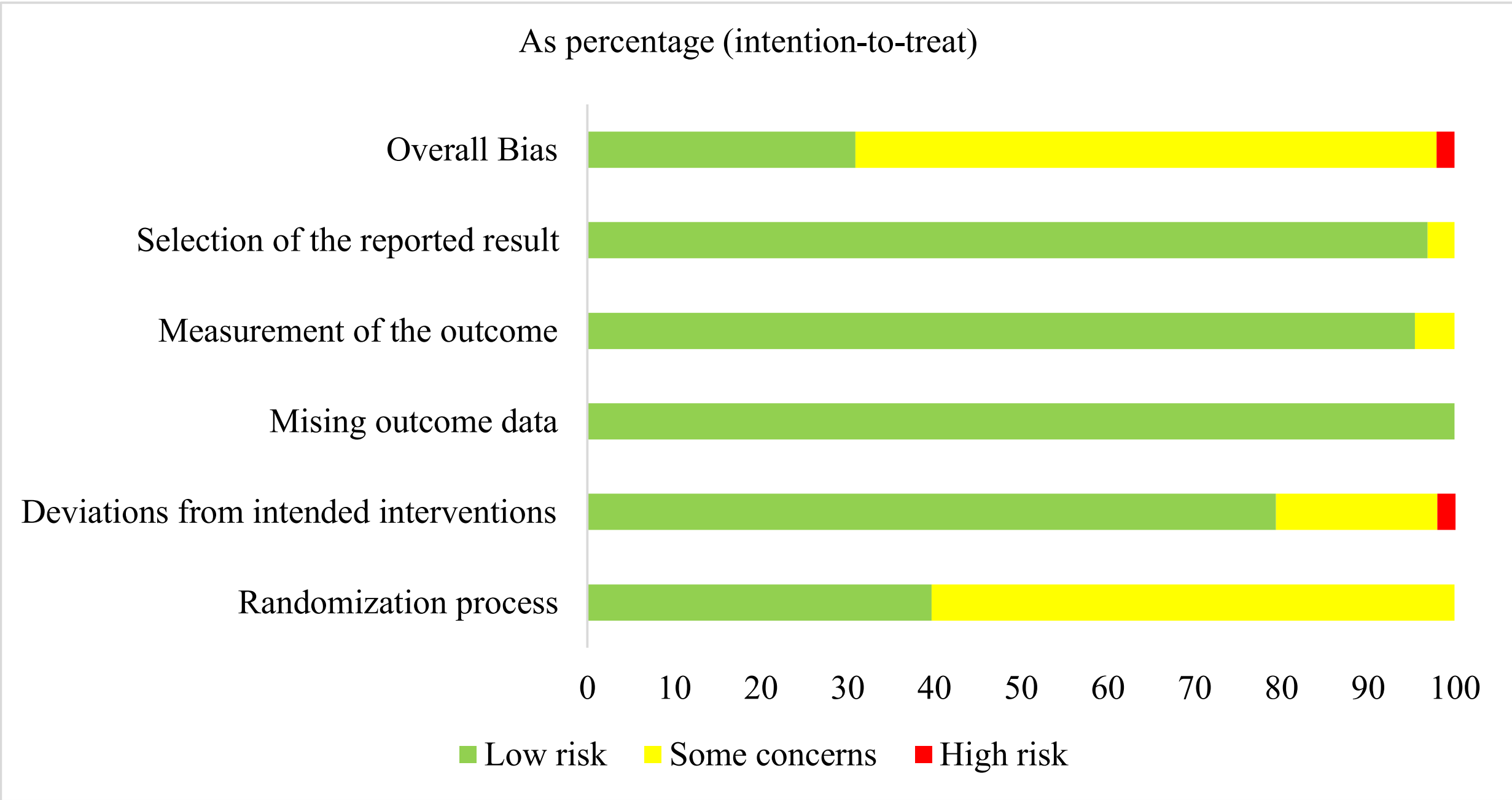
As percentage (intention-to-treat)
Overall Bias
Selection of the reported result
Measurement of the outcome
Mising outcome data
Deviations from intended interventions
Randomization process
0
10
20
30
40
50
60
70
80
90
100
Low risk
Some concerns
High risk
