# Supplementary material for: Rash associated with epidermal growth factor receptor inhibitors: a systematic review and meta-analysis of 194 randomized controlled trials
Source: Front Immunol. 2026 Aug 21;17:1814337. doi: 10.3389/fimmu.2026.1814337 (PMC13541753; doi:10.3389/fimmu.2026.1814337)
Supplement: Supplementary file 2 [file DataSheet2.pdf]

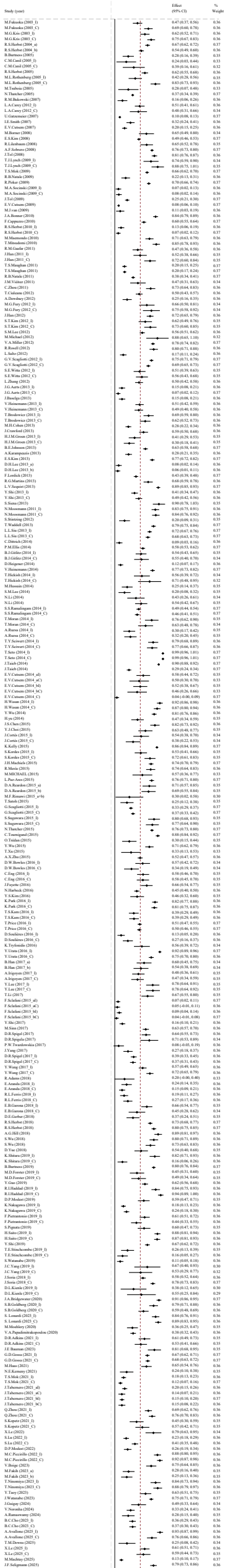

NOTE: Weights are from random-effects model
