# Supplementary material for: Rash associated with epidermal growth factor receptor inhibitors: a systematic review and meta-analysis of 194 randomized controlled trials
Source: Front Immunol. 2026 Aug 21;17:1814337. doi: 10.3389/fimmu.2026.1814337 (PMC13541753; doi:10.3389/fimmu.2026.1814337)
Supplement: Supplementary file 3 [file DataSheet3.pdf]

Study (year)	Risk ratio (95% CI)	% Weight
M.Fukuoka (2005)	0.68 (0.53, 0.86)	0.66
M.G.Kris (2003)	0.83 (0.69, 1.00)	0.68
R.S.Herbst (2004_a)	1.80 (1.46, 2.21)	0.67
R.S.Herbst (2004_b)	1.44 (1.16, 1.79)	0.67
B.Burtness (2005)	33.00 (2.03, 537.41)	0.11
C.M.Camil (2005)	0.61 (0.22, 1.70)	0.40
R.S.Herbst (2005)	2.21 (1.74, 2.82)	0.66
M.L.Rothenberg (2005)	0.50 (0.36, 0.71)	0.64
M.Tsuboa (2005)	1.85 (0.51, 6.67)	0.32
N.Thatcher (2005)	3.68 (2.84, 4.77)	0.66
R.M.Bukowski (2007)	17.65 (1.05, 298.15)	0.11
L.A.Carey (2012)	1.05 (0.70, 1.59)	0.62
U.Gatzemeier (2007)	14.97 (5.48, 40.93)	0.41
I.E.Smith (2007)	3.04 (1.56, 5.95)	0.53
E.V.Cutsem (2007)	23.50 (5.77, 95.68)	0.29
M.Borner (2008)	12.00 (3.05, 47.16)	0.30
K.S.Kim (2008)	4.84 (3.85, 6.08)	0.67
R.Lilenbaum (2008)	11.12 (3.64, 33.91)	0.37
A.F.Sobrero (2008)	15.59 (11.03, 22.03)	0.64
J.Tol (2008)	22.87 (11.01, 47.48)	0.51
T.J.Lynch (2009)	0.84 (0.65, 1.09)	0.66
S.Mok (2009)	2.96 (2.52, 3.47)	0.68
R.B.Natale (2009)	0.71 (0.43, 1.19)	0.59
R.Pirker (2009)	9.33 (6.94, 12.54)	0.65
M.A.Socinski (2009)	0.89 (0.31, 2.53)	0.39
J.Tol (2009)	46.50 (11.54, 187.29)	0.30
E.V.Cutsem (2009)	97.32 (6.02, 1574.67)	0.11
M.J.van (2009)	13.00 (0.75, 225.20)	0.10
J.A.Bonner (2010)	8.45 (5.60, 12.73)	0.62
F.Cappuzzo (2010)	7.97 (5.72, 11.12)	0.64
R.S.Herbst (2010)	1.80 (0.78, 4.17)	0.47
M.Maemondo (2010)	3.21 (2.23, 4.63)	0.63
T.Mitsudomi (2010)	10.69 (5.22, 21.88)	0.51
R.M.Gaafar (2011)	3.68 (2.03, 6.68)	0.56
J.Han (2011)	0.72 (0.53, 0.98)	0.65
T.S.Maughan (2011)	112.20 (6.97, 1806.96)	0.11
T.S.Maughan (2011)	108.40 (15.19, 773.74)	0.19
R.B.Natale (2011)	1.36 (1.16, 1.60)	0.68
J.M.Vicitner (2011)	2.57 (1.22, 5.43)	0.50
C.Zhou (2011)	3.78 (2.32, 6.15)	0.59
T.Ciuleanu (2012)	10.65 (5.72, 19.82)	0.54
A.Dowdney (2012)	20.49 (2.82, 148.82)	0.19
M.G.Fury (2012)	0.88 (0.63, 1.22)	0.63
J.Han (2012)	5.42 (3.57, 8.25)	0.62
S.T.Kim (2012)	0.86 (0.65, 1.13)	0.66
S.M.Lee (2012)	3.83 (2.89, 5.08)	0.66
M.Michael (2012)	16.67 (1.10, 252.34)	0.11
V.A.Miller (2012)	4.92 (3.55, 6.82)	0.64
R.Rosell (2012)	16.35 (6.25, 42.78)	0.42
L.Saltz (2012)	20.48 (2.80, 149.82)	0.18
G.V.Scagliotti (2012)	1.09 (1.01, 1.19)	0.69
S.E.Witta (2012)	0.91 (0.66, 1.27)	0.64
L.Zhang (2012)	5.23 (3.11, 8.87)	0.58
J.G.Aerts (2013)	2.11 (0.95, 4.69)	0.48
J.Baselga (2013)	17.65 (1.08, 288.36)	0.11
V.Heinemann (2013)	1.03 (0.81, 1.32)	0.66
T.Bredowicz (2013)	1.11 (0.88, 1.40)	0.67
M.H.Cohen (2013)	14.97 (5.54, 40.45)	0.41
J.Crawford (2013)	3.54 (1.91, 6.55)	0.55
J.H.M.Groen (2013)	1.37 (0.85, 2.21)	0.60
B.E.Johnson (2013)	2.81 (2.29, 3.45)	0.67
A.Karampeazis (2013)	95.00 (5.90, 1528.39)	0.11
E.S.Kim (2013)	36.95 (16.70, 81.77)	0.48
D.H.Lee (2013_a)	6.67 (0.39, 115.38)	0.10
D.H.Lee (2013_b)	5.11 (0.29, 90.08)	0.10
M.Lordick (2013)	8.25 (5.46, 12.44)	0.62
R.G.Martins (2013)	6.57 (3.60, 12.00)	0.55
L.V.Sequist (2013)	14.13 (6.89, 28.98)	0.51
Y.Shi (2013)	0.82 (0.66, 1.02)	0.67
S.Siena (2013)	4.75 (1.95, 11.60)	0.45
N.Moosmann (2011)	0.99 (0.87, 1.13)	0.68
S.Stintzing (2012)	19.35 (1.17, 321.20)	0.11
T.Waddell (2013)	8.44 (5.79, 12.32)	0.63
L.L.Siu (2013)	1.05 (0.96, 1.16)	0.69
C.Dittrich (2014)	7.64 (0.96, 60.71)	0.17
P.M.Ellis (2014)	7.65 (4.88, 12.02)	0.61
B.J.Gitlitz (2014)	0.98 (0.70, 1.39)	0.64
D.Heigener (2014)	32.33 (1.96, 532.25)	0.11
V.Heinemann (2014)	9.93 (6.68, 14.78)	0.62
T.Hickish (2014)	0.78 (0.50, 1.21)	0.61
M.Hussain (2014)	14.98 (0.93, 241.77)	0.11
S.M.Lee (2014)	4.00 (0.90, 17.68)	0.27
N.Li (2014)	6.50 (1.60, 26.36)	0.29
N.Li (2014)	8.39 (3.16, 22.24)	0.42
S.S.Ramalingam (2014)	1.07 (0.93, 1.23)	0.68
T.Moran (2014)	1.20 (0.88, 1.62)	0.65
A.Ruzsa (2014)	0.92 (0.52, 1.63)	0.57
T.Y.Seiwert (2014)	1.03 (0.85, 1.24)	0.67
T.Seto (2014)	1.00 (0.96, 1.04)	0.69
J.Taieb (2014)	29.00 (19.70, 42.69)	0.63
J.Taieb (2014)	218.84 (13.65, 3508.30)	0.11
E.V.Cutsem (2014_a)	1.17 (0.73, 1.86)	0.60
E.V.Cutsem (2014_b)	1.14 (0.68, 1.91)	0.58
E.V.Cutsem (2014)	0.13 (0.01, 2.51)	0.10
H.Wasan (2014)	1.06 (0.96, 1.18)	0.69
Y.Wu (2014)	9.13 (5.03, 16.54)	0.56
H.yu (2014)	4.58 (2.04, 10.26)	0.48
J.S.Chen (2015)	24.68 (6.29, 96.87)	0.30
Y.J.Choi (2015)	13.50 (3.42, 53.27)	0.30
J.Cortés (2015)	1.44 (0.88, 2.37)	0.59
K.Kelly (2015)	2.69 (2.30, 3.15)	0.68
S.Kordes (2015)	0.74 (0.56, 0.98)	0.66
J.H.Machiels (2015)	9.15 (5.42, 15.47)	0.58
R.Mesía (2015)	46.34 (6.60, 325.23)	0.19
M.MICHAEL (2015)	8.76 (2.19, 35.09)	0.30
L.Paz-Ares (2015)	4.82 (3.70, 6.28)	0.66
D.A.Reardon (2015_a)	3.50 (0.19, 64.67)	0.10
D.A.Reardon (2015_b)	4.50 (0.25, 79.53)	0.10
M.F.Rimawi (2015_a+b)	5.70 (0.68, 47.95)	0.17
T.Satoh (2015)	5.25 (1.23, 22.50)	0.28
G.Scagliotti (2015)	0.89 (0.75, 1.05)	0.68
S.Sugawara (2015)	1.05 (0.83, 1.32)	0.67
N.Thatcher (2015)	7.50 (5.81, 9.67)	0.66
C.Tournigand (2015)	10.40 (6.74, 16.03)	0.61
O.Trédan (2015)	5.95 (1.41, 25.07)	0.28
Y.Wu (2015)	6.70 (3.79, 11.87)	0.56
T.Xu (2015)	16.36 (0.99, 270.08)	0.11
A.X.Zhu (2015)	1.30 (1.10, 1.53)	0.68
D.W.Bowles (2016)	1.67 (1.00, 2.79)	0.59
C.Eng (2016)	1.01 (0.74, 1.37)	0.65
J.Fayette (2016)	1.33 (0.97, 1.83)	0.65
N.Harbeck (2016)	6.93 (3.87, 12.42)	0.56
Y.S.Kim (2016)	4.31 (1.78, 10.43)	0.45
K.Park (2016)	1.02 (0.92, 1.13)	0.69
T.S.Kam (2016)	0.99 (0.69, 1.42)	0.63
T.Price (2016)	1.02 (0.90, 1.15)	0.68
D.Soulières (2016)	0.49 (0.24, 0.97)	0.52
K.Tryfonidis (2016)	3.24 (1.48, 7.10)	0.48
Y.Urata (2016)	1.24 (1.15, 1.33)	0.69
B.Han (2017_a)	6.00 (1.57, 22.89)	0.31
B.Han (2017_b)	10.73 (1.56, 74.04)	0.19
A.Irigoyen (2017)	1.04 (0.71, 1.52)	0.63
Y.Lee (2017)	1.00 (0.77, 1.28)	0.66
T.Li (2017)	2.60 (1.33, 5.05)	0.53
F.Sclafani (2015_a)	1.30 (0.36, 4.72)	0.32
F.Sclafani (2015_b)	2.63 (0.60, 11.49)	0.28
Y.Shi (2017)	10.65 (2.56, 44.31)	0.29
M.Sinn (2017)	15.85 (2.12, 118.51)	0.18
D.R.Spigel (2017)	11.76 (3.88, 35.67)	0.37
D.R.Spigela (2017)	1.24 (0.82, 1.87)	0.62
P.W.Twardowskia (2017)	5.00 (0.25, 99.16)	0.10
J.Yang (2017)	0.86 (0.53, 1.40)	0.59
D.R.Spigel (2017)	1.04 (0.83, 1.30)	0.67
Y.Wang (2017)	0.79 (0.67, 0.94)	0.68
R.Adams (2018)	7.44 (0.42, 132.95)	0.10
E.Aranda (2018)	1.62 (0.88, 2.96)	0.55
R.L.Ferris (2018)	0.71 (0.41, 1.24)	0.57
E.B.Garona (2018)	1.44 (0.96, 2.18)	0.62
D.E.Gerber (2018)	4.19 (1.54, 11.40)	0.41
R.S.Herbst (2018)	10.60 (7.22, 15.55)	0.63
R.S.Herbst (2018)	6.90 (4.93, 9.65)	0.64
A.G.Hill (2018)	1.12 (0.97, 1.31)	0.68
S.Wu (2018)	12.64 (5.37, 29.73)	0.46
S.Wu (2018)	8.04 (3.92, 16.50)	0.51
D.Yue (2018)	47.45 (2.98, 755.49)	0.11
K.Shitara (2019)	5.23 (2.73, 10.01)	0.54
B.Burtness (2019)	3.74 (2.86, 4.88)	0.66
M.D.Forster (2019)	0.93 (0.60, 1.45)	0.61
Y.Guo (2019)	5.85 (3.31, 10.32)	0.57
R.I.Haddad (2019)	0.89 (0.79, 1.00)	0.68
D.P.Modest (2019)	19.59 (2.81, 136.44)	0.19
K.Nakagawa (2019)	0.74 (0.51, 1.06)	0.63
F.Pietrantonio (2019)	1.38 (1.02, 1.86)	0.65
S.Pignata (2019)	31.20 (4.43, 219.95)	0.19
H.Saito (2019)	1.01 (0.91, 1.11)	0.69
Y.Shi (2019)	12.26 (6.47, 23.24)	0.54
T.E.Stinchcombe (2019)	1.65 (0.70, 3.84)	0.46
S.Watanabe (2019)	1.44 (0.58, 3.63)	0.44
J.C.Yang (2019)	1.26 (0.69, 2.30)	0.55
J.Soria (2018)	0.74 (0.66, 0.83)	0.69
D.L.Kienle (2019)	0.71 (0.29, 1.69)	0.45
J.A.Bridgewater (2020)	4.66 (3.29, 6.62)	0.64
S.B.Goldberg (2020)	1.35 (1.09, 1.67)	0.67
S. Lonardi (2023)	0.94 (0.84, 1.06)	0.69
M.Moehlery (2020)	8.43 (2.67, 26.58)	0.36
V.A.Papadimitrakopoulou (2020)	5.69 (2.97, 10.88)	0.54
D.R.Adkins (2021)	1.14 (0.84, 1.55)	0.65
J.E Bauman (2023)	7.04 (2.40, 20.68)	0.38
G.D.Gossa (2021)	0.99 (0.90, 1.09)	0.69
M.Haas (2021)	12.34 (3.17, 48.02)	0.30
N.E.Kemeny (2021)	19.50 (1.18, 323.41)	0.11
T.S.Mok (2021)	1.56 (0.99, 2.45)	0.60
J.Tabernero (2021_a)	1.40 (0.81, 2.44)	0.57
J.Tabernero (2021_b)	1.02 (0.57, 1.81)	0.56
Q.Zhou (2021)	0.90 (0.78, 1.03)	0.68
S.Kopetz (2021)	0.79 (0.53, 1.19)	0.62
X.Le (2022)	1.58 (1.04, 2.42)	0.62
S.Lu (2022)	0.56 (0.42, 0.75)	0.65
D.P.Modest (2022)	4.06 (1.95, 8.43)	0.50
M.C.Piccirillo (2022)	0.95 (0.85, 1.05)	0.69
V.Boige (2023)	17.91 (5.86, 54.70)	0.37
M.Fakih (2023_a)	7.36 (1.03, 52.72)	0.19
M.Fakih (2023_b)	13.00 (0.80, 210.31)	0.11
T.Ninomiya (2023)	0.95 (0.81, 1.12)	0.68
Y.Taoy (2023)	6.94 (3.16, 15.22)	0.48
J.Watanabe (2023)	23.40 (13.67, 40.07)	0.58
J.Guigay (2024)	10.00 (2.50, 40.05)	0.30
V.Noronha (2024)	1.82 (1.15, 2.87)	0.60
A.Ramaswamy (2024)	27.00 (1.65, 441.43)	0.11
B.C.Cho (2023)	0.99 (0.76, 1.29)	0.66
A.Avallone (2025)	1.22 (1.05, 1.41)	0.68
T.M.Downs (2025)	7.28 (0.44, 119.84)	0.11
X.Le (2025)	1.04 (0.78, 1.40)	0.65
M.Machtay (2025)	78.19 (4.83, 1266.00)	0.11
J.F.Seligmann (2025)	14.16 (6.86, 29.22)	0.51
A.Stahler (2024)	0.87 (0.45, 1.66)	0.54
Overall, DL (I^2 = 95.7%, p < 0.001)	2.62 (2.37, 2.90)	100.00

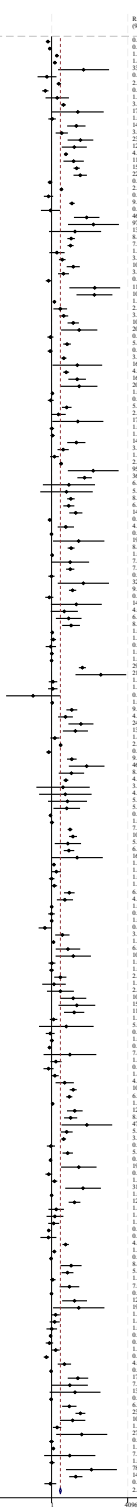

NOTE: Weights are from random-effects model; continuity correction applied to studies with zero cells
