# Supplementary material for: Rash associated with epidermal growth factor receptor inhibitors: a systematic review and meta-analysis of 194 randomized controlled trials
Source: Front Immunol. 2026 Aug 21;17:1814337. doi: 10.3389/fimmu.2026.1814337 (PMC13541753; doi:10.3389/fimmu.2026.1814337)
Supplement: Supplementary file 4 [file DataSheet4.pdf]

all-grade

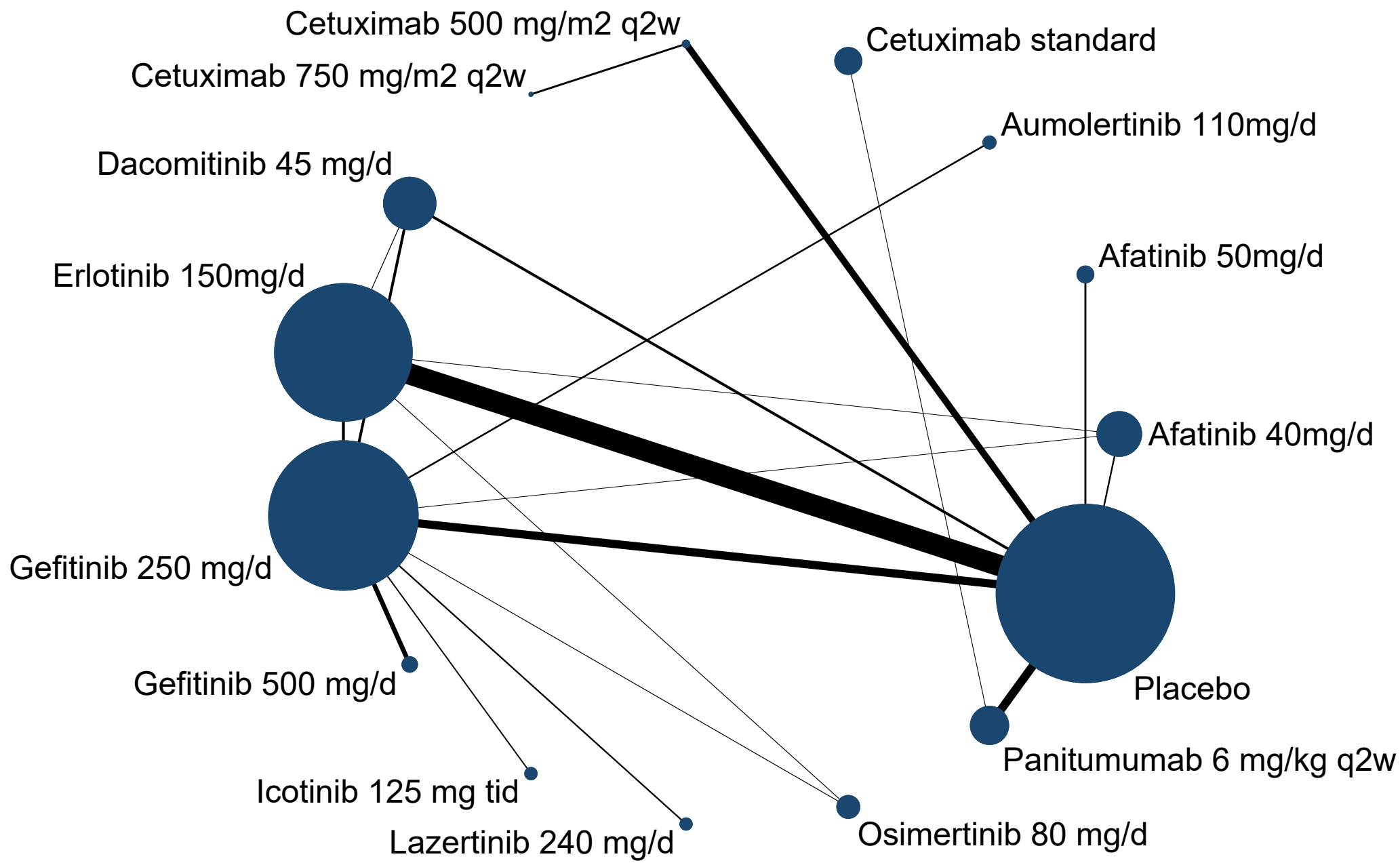

Cetuximab 500 mg/m2 q2w
Cetuximab 750 mg/m2 q2w
Cetuximab standard
Aumolertinib 110mg/d
Dacomitinib 45 mg/d
Afatinib 50mg/d
Erlotinib 150mg/d
Afatinib 40mg/d
Gefitinib 250 mg/d
Placebo
Gefitinib 500 mg/d
Panitumumab 6 mg/kg q2w
Icotinib 125 mg tid
Lazertinib 240 mg/d
Osimertinib 80 mg/d
