# Supplementary material for: Rash associated with epidermal growth factor receptor inhibitors: a systematic review and meta-analysis of 194 randomized controlled trials
Source: Front Immunol. 2026 Aug 21;17:1814337. doi: 10.3389/fimmu.2026.1814337 (PMC13541753; doi:10.3389/fimmu.2026.1814337)
Supplement: Supplementary file 5 [file DataSheet5.pdf]

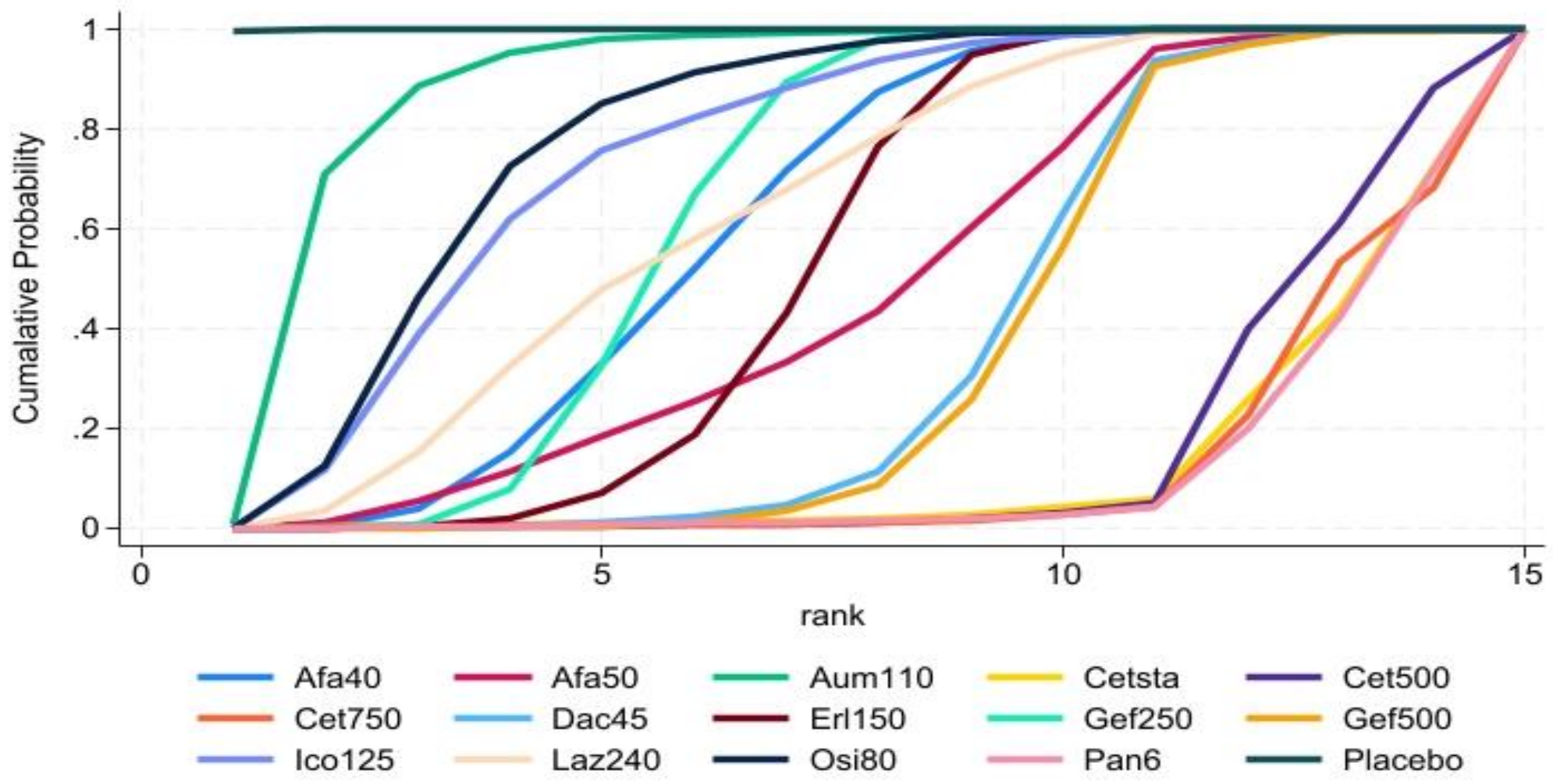

Treatmt	SUCRA
Pan6	10.5
Cet750	11.1
Cetsta	11.5
Cet500	14.4
Gef500	34.6
Dac45	35.9
Afa50	47.8
Erl150	52.9
Afa40	61.3
Laz240	63.2
Gef250	64
Ico125	74.9
Osi80	78.5
Aum110	89.4
Placebo	100
