# Supplementary material for: Rash associated with epidermal growth factor receptor inhibitors: a systematic review and meta-analysis of 194 randomized controlled trials
Source: Front Immunol. 2026 Aug 21;17:1814337. doi: 10.3389/fimmu.2026.1814337 (PMC13541753; doi:10.3389/fimmu.2026.1814337)
Supplement: Supplementary file 6 [file DataSheet6.pdf]

All-grade rash RR(95% CI)

Afa40	1.22 (0.63,2.33)	0.55 (0.29,1.03)	**5.71 (1.17,27.87)**	**4.43 (1.27,15.45)**	**5.05 (1.28,19.95)**	1.42 (0.91,2.20)	1.09 (0.80,1.48)	0.97 (0.71,1.33)	1.45 (0.93,2.24)	0.80 (0.44,1.45)	0.96 (0.52,1.78)	0.76 (0.46,1.26)	**5.81 (1.28,26.36)**	**0.25 (0.18,0.34)**
0.82 (0.43,1.58)	Afa50	0.45 (0.20,1.03)	4.69 (0.90,24.51)	3.64 (0.96,13.82)	4.16 (0.97,17.71)	1.17 (0.59,2.30)	0.90 (0.49,1.65)	0.80 (0.43,1.48)	1.19 (0.60,2.37)	0.66 (0.30,1.47)	0.79 (0.35,1.79)	0.63 (0.30,1.31)	4.78 (0.98,23.25)	**0.20 (0.12,0.36)**
1.82 (0.97,3.41)	2.22 (0.97,5.05)	Aum110	**10.40 (1.97,54.94)**	**8.07 (2.09,31.07)**	**9.21 (2.13,39.78)**	2.58 (1.34,4.98)	1.98 (1.10,3.57)	**1.77 (1.03,3.06)**	2.63 (1.41,4.92)	1.46 (0.69,3.08)	1.76 (0.82,3.76)	1.39 (0.69,2.78)	**10.58 (2.15,52.14)**	**0.45 (0.25,0.82)**
**0.18 (0.04,0.86)**	0.21 (0.04,1.11)	**0.10 (0.02,0.51)**	Cetsta	0.78 (0.11,5.55)	0.89 (0.11,6.86)	0.25 (0.05,1.23)	**0.19 (0.04,0.92)**	**0.17 (0.04,0.82)**	0.25 (0.05,1.26)	**0.14 (0.03,0.73)**	**0.17 (0.03,0.89)**	**0.13 (0.03,0.68)**	1.02 (0.63,1.64)	**0.04 (0.01,0.20)**
**0.23 (0.06,0.79)**	0.27 (0.07,1.04)	**0.12 (0.03,0.48)**	1.29 (0.18,9.22)	Cet500	1.14 (0.65,2.02)	0.32 (0.09,1.13)	**0.25 (0.07,0.84)**	**0.22 (0.06,0.75)**	0.33 (0.09,1.16)	**0.18 (0.05,0.69)**	**0.22 (0.06,0.83)**	**0.17 (0.05,0.63)**	1.31 (0.19,8.85)	**0.06 (0.02,0.19)**
**0.20 (0.05,0.78)**	0.24 (0.06,1.03)	**0.11 (0.03,0.47)**	1.13 (0.15,8.76)	0.88 (0.50,1.55)	Cet750	0.28 (0.07,1.12)	**0.22 (0.06,0.84)**	**0.19 (0.05,0.75)**	0.29 (0.07,1.15)	**0.16 (0.04,0.68)**	**0.19 (0.04,0.82)**	**0.15 (0.04,0.62)**	1.15 (0.16,8.42)	**0.05 (0.01,0.19)**
0.71 (0.45,1.09)	0.86 (0.44,1.69)	**0.39 (0.20,0.75)**	4.02 (0.82,19.87)	3.12 (0.88,11.05)	3.56 (0.89,14.25)	Dac45	0.77 (0.54,1.09)	0.69 (0.48,0.99)	1.02 (0.63,1.64)	0.56 (0.30,1.06)	0.68 (0.36,1.29)	**0.54 (0.31,0.92)**	4.10 (0.89,18.80)	**0.17 (0.12,0.25)**
0.92 (0.68,1.25)	1.12 (0.61,2.06)	**0.50 (0.28,0.91)**	**5.24 (1.09,25.20)**	**4.07 (1.19,13.91)**	**4.64 (1.20,18.00)**	1.30 (0.92,1.84)	Erl150	0.89 (0.72,1.11)	1.33 (0.91,1.94)	0.73 (0.42,1.28)	0.89 (0.50,1.57)	0.70 (0.45,1.08)	**5.34 (1.20,23.82)**	**0.23 (0.18,0.29)**
1.03 (0.75,1.40)	1.25 (0.67,2.32)	**0.56 (0.33,0.97)**	**5.87 (1.22,28.29)**	**4.55 (1.33,15.63)**	**5.20 (1.34,20.21)**	1.46 (1.01,2.10)	1.12 (0.90,1.40)	Gef250	**1.49 (1.10,2.02)**	0.82 (0.49,1.37)	0.99 (0.58,1.68)	0.78 (0.51,1.21)	**5.97 (1.33,26.74)**	**0.25 (0.20,0.33)**
0.69 (0.45,1.07)	0.84 (0.42,1.68)	**0.38 (0.20,0.71)**	3.95 (0.79,19.60)	3.06 (0.86,10.92)	3.49 (0.87,14.07)	0.98 (0.61,1.58)	0.75 (0.52,1.10)	**0.67 (0.50,0.91)**	Gef500	0.55 (0.30,1.00)	0.67 (0.36,1.23)	**0.53 (0.31,0.89)**	4.02 (0.87,18.55)	**0.17 (0.12,0.25)**
1.25 (0.69,2.27)	1.52 (0.68,3.39)	0.69 (0.33,1.45)	**7.14 (1.37,37.30)**	**5.54 (1.46,21.04)**	**6.32 (1.48,26.97)**	1.77 (0.95,3.32)	1.36 (0.78,2.37)	1.22 (0.73,2.03)	1.81 (1.00,3.28)	Ico125	1.21 (0.58,2.52)	0.95 (0.49,1.86)	**7.26 (1.49,35.38)**	**0.31 (0.18,0.55)**
1.04 (0.56,1.92)	1.26 (0.56,2.85)	0.57 (0.27,1.22)	**5.92 (1.13,31.14)**	**4.59 (1.20,17.59)**	**5.24 (1.22,22.53)**	1.47 (0.77,2.80)	1.13 (0.64,2.01)	1.01 (0.59,1.71)	1.50 (0.81,2.77)	0.83 (0.40,1.73)	Laz240	0.79 (0.40,1.56)	**6.03 (1.23,29.55)**	**0.26 (0.14,0.46)**
1.31 (0.79,2.18)	1.60 (0.77,3.33)	0.72 (0.36,1.44)	**7.50 (1.48,37.95)**	**5.81 (1.59,21.24)**	**6.64 (1.61,27.31)**	**1.86 (1.09,3.19)**	1.43 (0.93,2.20)	1.28 (0.83,1.97)	**1.90 (1.12,3.22)**	1.05 (0.54,2.05)	1.27 (0.64,2.51)	Osi80	**7.63 (1.62,35.95)**	**0.32 (0.20,0.52)**
**0.17 (0.04,0.78)**	0.21 (0.04,1.02)	**0.09 (0.02,0.47)**	0.98 (0.61,1.58)	0.76 (0.11,5.14)	0.87 (0.12,6.37)	0.24 (0.05,1.12)	**0.19 (0.04,0.84)**	**0.17 (0.04,0.75)**	0.25 (0.05,1.15)	**0.14 (0.03,0.67)**	**0.17 (0.03,0.81)**	**0.13 (0.03,0.62)**	Pan6	**0.04 (0.01,0.19)**
**4.05 (2.93,5.58)**	**4.92 (2.79,8.66)**	**2.22 (1.22,4.04)**	**23.09 (4.89,109.12)**	**17.91 (5.35,59.95)**	**20.44 (5.38,77.69)**	**5.74 (3.96,8.32)**	**4.40 (3.49,5.56)**	**3.93 (3.07,5.05)**	**5.85 (3.94,8.69)**	**3.24 (1.83,5.71)**	**3.90 (2.17,7.01)**	**3.08 (1.93,4.92)**	**23.50 (5.36,103.01)**	Placebo
